# Circulating Extracellular Vesicles in Alcoholic Liver Disease Affect Skeletal Muscle Homeostasis and Differentiation

**DOI:** 10.1002/jcsm.13675

**Published:** 2025-02-07

**Authors:** Laura Barberi, Cristiana Porcu, Caterina Boccia, Marianna Cosentino, Carmine Nicoletti, Barbara Peruzzi, Francesca Iosi, Flavia Forconi, Giulia Bagnato, Gabriella Dobrowolny, Simone Di Cola, Lucia Lapenna, Gianluca Cera, Manuela Merli, Antonio Musarò

**Affiliations:** ^1^ DAHFMO‐Unit of Histology and Medical Embryology Sapienza University of Rome, Laboratory Affiliated to Istituto Pasteur Italia – Fondazione Cenci Bolognetti Rome Italy; ^2^ Bone Pathophysiology Research Unit Bambino Gesù Children's Hospital, IRCCS Rome Italy; ^3^ Core Facilities, Microscopy Area Istituto Superiore di Sanità Rome Italy; ^4^ Department of Translational and Precision Medicine Sapienza University of Rome Rome Italy; ^5^ Department of Orthopaedics and Traumatology Policlinico Umberto I Rome Italy; ^6^ Scuola Superiore di Studi Avanzati Sapienza (SSAS) Sapienza University of Rome Rome Italy

**Keywords:** alcoholic liver disease (ALD), extracellular vesicles (EVs), liver‐muscle interplay, microRNA (miRNA), muscle atrophy, sarcopenia

## Abstract

**Background:**

The mechanisms underlying muscle alteration associated to alcoholic liver disease (ALD) are not fully understood and the physiopathologic mediators of the liver–muscle interplay remains elusive. We investigated the role of circulating extracellular vesicles (EVs) in ALD as potential mediators of muscle atrophy.

**Methods:**

We established a mouse model of sarcopenia associated to ALD, by feeding mice with an alcoholic diet for 8 weeks. We investigated the effects of hepatic and circulating EVs isolated from these mice (EtOH mice; *n* = 7 females) on muscle cell cultures, comparing them with EVs from mice fed with a standard diet (CD mice; *n* = 6 females). Additionally, we examined the impact of circulating EVs from patients with alcohol‐related cirrhosis (7 males and 2 females, mean age 55.4 years) on primary human muscle cells, comparing them with EVs from age‐matched healthy subjects (6 males and 3 females). We analysed the miRNA profile of the EVs to identify potential mediators of ALD‐associated sarcopenia.

**Results:**

We demonstrated that circulating EVs were internalized by muscle cells and that EVs from ALD mice and cirrhotic patients caused alteration in the myogenic program. Molecular analysis revealed that serum EVs from ALD mice reduced protein synthesis in C2C12 cells, decreasing levels of p‐AKT/AKT (−54.6%; *p* < 0.05), p‐mTOR/mTOR (−54.5%; *p* < 0.05) and p‐GSK3(Ser9)/GSK3 (−30.63%). Similarly, hepatic EVs induced defects in muscle differentiation, with reduced levels of p‐AKT/AKT (−39.1%; *p* < 0.05), p‐mTOR/mTOR (−30.1%; *p* < 0.05) and p‐GSK3(Ser9)/GSK3 (−40%). C2C12 cells treated with either serum or hepatic EtOH‐EVs exhibited upregulated expression of muscle‐specific atrophy markers Atrogin‐1 (+61.2% and +189.5%, respectively; *p* < 0.05) and MuRF1 (+260.4% and +112.5%, respectively; *p* < 0.05), along with an increased LC3‐II/‐I ratio (+131.5% and +40.2%, respectively; *p* < 0.05), indicating enhanced autophagy. MiRNA analysis revealed that both circulating and hepatic EVs from ALD mice showed elevated expression of miR‐21, miR‐155, miR‐223 and miR‐122 (+230% and +292%, respectively; *p* < 0.01) suggesting their potential role in sarcopenia.

Human muscle cells exposed to EVs from cirrhotic patients exhibited reduced protein synthesis and upregulated Atrogin‐1 (+113%; *p* < 0.05) and MuRF1 (+86.3%; *p* < 0.05), indicating proteasome activation. Circulating EVs of alcoholic patients showed upregulation of the same miRNAs observed in EtOH mice, including the liver‐specific miR‐122 (+260%; *p* < 0.05) suggesting, also in human liver disease, a hepatic origin of circulating EVs.

**Conclusions:**

Our study highlights the critical role of ALD‐derived circulating EVs in affecting muscle homeostasis and myogenic program, suggesting potential therapeutic targets for mitigating muscle loss in ALD.

## 1 Introduction

The loss of mass and strength, referred to as sarcopenia, is a highly prevalent condition in patients with chronic liver disease (CLD), associated with significant clinical complications, poor prognosis and high mortality [1, 2]. Severe sarcopenia occurs in approximately 60% of patients with alcoholic liver disease (ALD), the most common cause of liver disease globally [3].

Although various factors contribute to sarcopenia in liver disease, the imbalance between protein synthesis and degradation is recognized as the main cause of its pathogenesis [4, 5, 6]. The PI3K/AKT/mTOR pathway coordinates muscle protein synthesis and degradation, as well as myogenic differentiation, making its regulation crucial for muscle growth and homeostasis [7, 8]. Although many factors implicated in the development of sarcopenia in liver disease have been identified [4], the mediators of the liver–muscle axis have not yet been fully understood.

Extracellular vesicles (EVs), which are membrane‐enclosed particles released from nearly all cells, have emerged as a novel class of mediators of intercellular communication and inter‐organ crosstalk [9]. These vesicles carry proteins, lipids, RNA and DNA molecules modulating intracellular pathways and the biological activity of recipient cells [9, 10]. In the liver, EVs are produced by all hepatic cells to sustain liver functions, maintain hepatic homeostasis and promote cell survival and proliferation [11]. Changes in EV amount and composition significantly contribute to the pathogenesis and/or progression of liver diseases [12, 13, 14, 15]. These EVs are loaded with detrimental factors and specific microRNAs, which may be the main mediators of the effects of EVs [16, 17]. Notably, alcohol consumption itself induces a large release of EVs by miR‐155‐mediated suppression of autophagic functions [18, 19].

As the liver plays a central role in systemic homeostasis and, when injured, can induce pathogenic processes in remote organs through EVs released into circulation [19, 20], we speculate that in ALD, liver‐derived EVs could deliver damaging molecules, especially miRNAs, to skeletal muscle, inducing or contributing to sarcopenia. By exposing muscle cell culture to circulating EVs from a mouse model of ALD, we aimed to evaluate their effects on muscle differentiation and protein homeostasis. A similar analysis of hepatic interstitial EVs was performed to investigate their impact on skeletal muscle cells and to support the hypothesis of a hepatic origin of the circulating EVs mediating sarcopenia. Finally, we analysed the effects of circulating EVs isolated from ALD patients on the differentiation of primary human skeletal muscle cells.

## 2 Materials and Methods

### 2.1 Animal Model and Treatment

All procedures were conducted according to the guidelines of the Declaration of Helsinki, approved by Institutional Review Board of the animal facilities of DIEM and the Italian National Institute of Health (n° 609/2015‐PR; n° 864/2020‐PR) and reported following the ARRIVE guidelines. Mice were obtained from Charles River Laboratories. Fourteen‐week‐old female C57BL/6J mice and 24‐month‐old mice were housed in a temperature‐controlled room (22°C) with a 12:12 h light–dark cycle. Mice received a standard diet (CD mice; *n* = 6) or Lieber–DeCarli liquid diet (Dyets Inc., 710 260) containing 5% ethanol (EtOH mice; *n* = 7) daily for 8 weeks, with free access to food.

### 2.2 Patients

Blood samples were obtained from 9 control healthy subjects and 9 patients with alcohol‐related cirrhosis. Healthy subjects were recruited from blood donors, whereas cirrhotic patients were those hospitalized or followed in the outpatient clinic of Sapienza University Hospital Rome, Italy. All subjects gave informed consent for this procedure and data utilization. Serum was separated from cells by centrifugation for 10 min at 2000 rpm. Blood sampling was performed according to a protocol approved by the Ethical Committee of Sapienza University of Rome (Prot 0057/2023). The cirrhotic patients were 7 males and 2 females, with a mean age of 55.4 years (range 34–66 years). The diagnosis of cirrhosis was based on histologic and/or clinical evidence, including liver function test, imaging and endoscopic features of portal hypertension. Liver disease severity was evaluated through the MELD score (mean MELD score was 17.6 ± 5.7). The presence of sarcopenia was assessed through CT examination when available, and all patients were found to be sarcopenic.

### 2.3 Extracellular Vesicle Isolation From Serum and Liver Samples

EVs from mice and human serum samples were isolated with a precipitation method, whereas interstitial EVs from liver samples were isolated with an ultracentrifugation‐based method [21]. Both procedures are described in Supplementary Methods.

### 2.4 C2C12 Cell Culture and EVs Treatment

Murine C2C12 myoblast cells were cultured in growth medium (GM), containing DMEM supplemented with 10% FBS (Sigma‐Aldrich), and then shifted in differentiation medium (DM), containing DMEM supplemented with 2% horse serum (Sigma‐Aldrich). Cells were exposed to 50 μg/mL of EVs isolated from the serum and the liver of CD and EtOH mice at the time of their shift into differentiation medium (DM0) and after 3 days (DM3) and were analysed on the fifth day in DM (DM5).

### 2.5 Primary Human Myoblasts and Treatment

Primary human myoblasts were extracted from fresh muscle biopsies of adult healthy subjects undergoing orthopaedic surgery intervention, as detailed in the Supplementary Methods.

### 2.6 miRNA Cell Transfection

Mimic miR‐122 and miR155 miRNAs were transfected in C2C12 cells as detailed in the Supplementary Methods.

### 2.7 RNA Extraction and Real‐Time PCR

For RNA isolation from EVs, the total exosome RNA and protein isolation kit (Thermo Fisher) were used following the manufacturer's instructions. Total RNA from cell culture and tissue samples was extracted and analysed as described in [22, 23]. HPRT1 and miR‐26a were used to normalize mRNA and miRNA expression, respectively [24]. Data are expressed as fold induction of 2^−ΔΔCt^ mean ± SEM.

### 2.8 Protein Extraction and Western Blot

Protein expression analysis was performed as reported in [8] and in the Supplementary Methods.

Histologic and immunofluorescence analysis was performed as reported in [8] and detailed in the Supplementary Methods.

### 2.9 Alanine Aminotransferase Activity Assay

Blood sera were separated by centrifugation at 2000 rpm for 10 min and then assayed immediately or stored at −20°C. Alanine aminotransferase (ALT) activity was determined using a commercially available diagnostic kit (Sigma‐Aldrich), according to the manufacturer's instructions.

### 2.10 Functional Analysis on Mice

CD and EtOH mice force measurements were performed on extensor digitorum longus (EDL) muscle through the *ex vivo* methodology [25]. The EDL muscle was subjected to a 0.40 s pulse train with a frequency of 180 Hz to evaluate the tetanic force. Specific force was obtained normalizing isometric tetanic force by muscle cross‐sectional area (CSA).

### 2.11 Statistical Analysis

Statistical analysis was performed with GraphPad Prism 8.0 Software (GraphPad Software). All data are expressed as mean ± SEM. Groups were compared by non‐parametric Mann–Whitney *U* test. A value of <0.05 was considered statistically significant.

## 3 Results

### 3.1 Establishment of a Mouse Model of Liver Disease–Associated Sarcopenia

To generate an experimental model of sarcopenia associated to liver disease we fed mice with an alcoholic diet (Lieber–De Carli diet containing 5% ethanol) for 8 weeks inducing ALD (EtOH mice).

**FIGURE 1:**
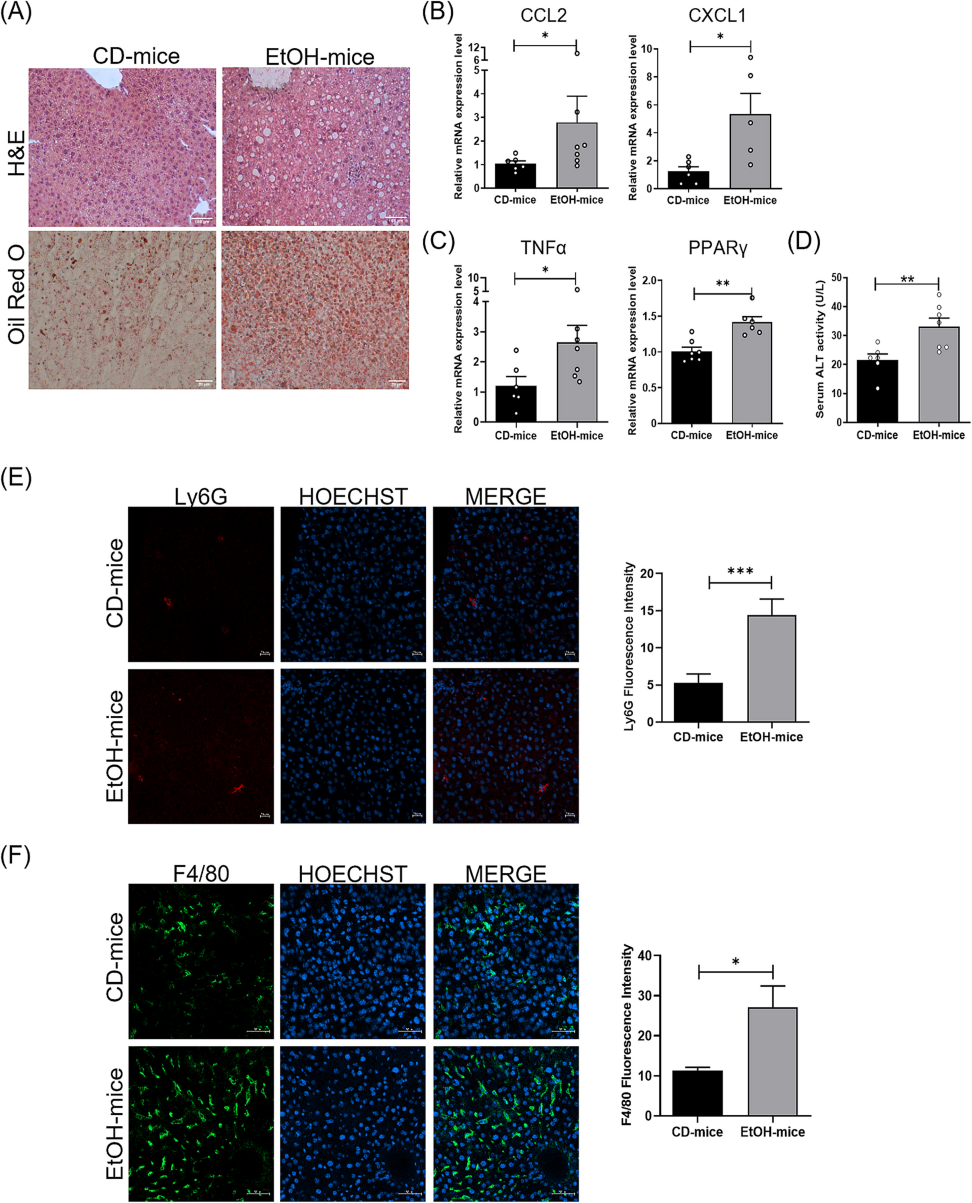
Hepatic steatosis and inflammatory activation in EtOH‐fed mice, compared to control diet (CD) mice. (A) Representative images of liver cross sections of CD mice and EtOH mice stained with haematoxylin and eosin (H&E) (upper panels; scale bar, 100 μm) and Oil red O (lower panels; scale bar, 20 μm) (*n* ≥ 3 per group). (B,C) Real‐time PCR analysis to evaluate mRNA expression levels of CCL‐2 (B, left panel), CXCL1 (B, right panel), TNF‐α (C, left panel) and PPAR‐γ (C, right panel) in liver tissue of CD and EtOH mice (*n* ≥ 6 per group). (D) Quantification of alanine aminotransferase (ALT) activity in serum samples of CD and EtOH mice (*n* ≥ 6 per group). (E,F) Representative images of immunofluorescence analysis of Ly6G (E, left panel) and F4/80 (F, left panel) positive cells (in green) in liver tissue of CD and EtOH mice (scale bar, 50 μm). Nuclei are stained with Hoechst (blue). Intensity of Ly6G and F4/80 fluorescent signals was quantified (mice per group = 4; image fields measured per mouse ≥ 3) (E,F, right panels, respectively). Nuclei are stained with Hoechst (blue). Data are expressed as mean ± SEM. Data were analysed by Mann–Whitney *U* test. EtOH mice versus CD mice, *p < 0.05. All data are represented as mean ± SEM. Data were analysed by Mann–Whitney *U* test. EtOH mice versus CD mice, *p < 0.05 and **p < 0.01.

We evaluated the development of liver disease examining liver tissue by histological and molecular analyses. Haematoxylin and eosin (H&E) staining revealed extensive hepatic steatosis along with an inflammatory cell infiltration in EtOH mice, compared with control diet (CD) mice (Figure 1A); lipid accumulation in the alcoholic mice was also revealed by Oil red O staining (Figure 1A).

Histological features were confirmed by real‐time PCR analyses, showing alcohol‐induced upregulation of the chemokines MCP‐1/CCL‐2 and CXCL1 (Figure 1B), along with the cytokine TNF‐α (Figure 1C), implicated in hepatic inflammation [26], and of PPARγ (Figure 1C) that promotes both hepatic inflammation and steatosis [27]. Consistent with previous studies [S1], chronic feeding of mice with Lieber–DeCarli diet induced a statistically significant increase in serum alanine aminotransferase (ALT), compared to CD mice (Figure 1D).

In line with the role of CXCL1 in mediating neutrophil and macrophage recruitment [S2, S3], we also observed a significant increase in inflammatory cell infiltration in the liver of EtOH mice, compared to CD mice, as evidenced by elevated expression levels of Ly6C and F4/80 (Figure 1E,F).

**FIGURE 2:**
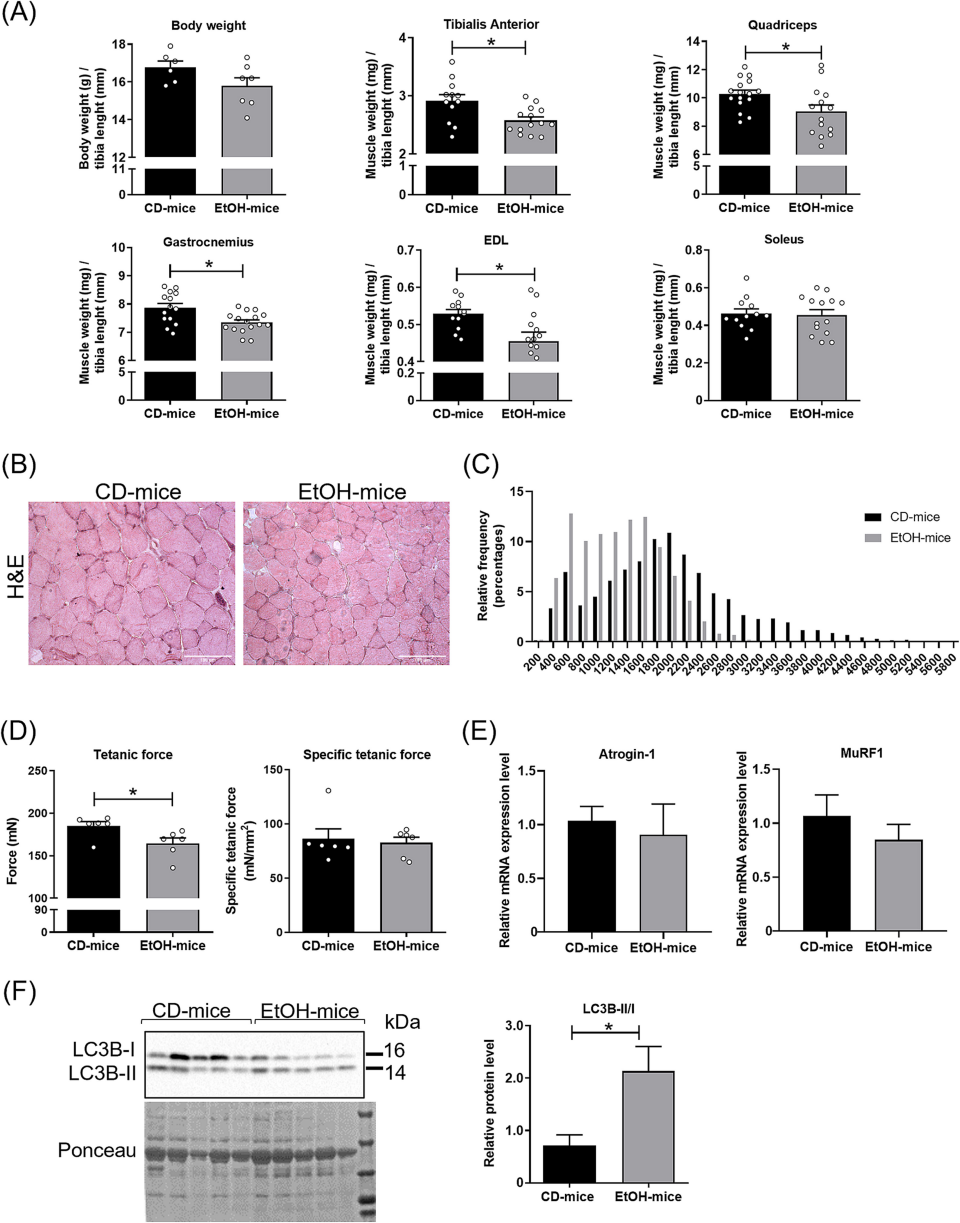
Muscle atrophy in EtOH mice compared with CD mice. (A) Body weight (*n* ≥ 6 per group) and muscle mass of different skeletal muscles (*n* ≥ 12 per group) of CD and EtOH mice. Muscle weights (mg) were normalized to tibia length (mm). (B) Representative images of cross sections of extensor digitorum longus (EDL) muscle fibres from CD and EtOH mice stained with haematoxylin and eosin (H&E) (scale bar, 100 μm) (*n* ≥ 3 per group). (C) Frequency distribution of cross‐sectional area (CSA) of EDL muscle fibres (*n* ≥ 3 per group). (D) Tetanic force (left panel) and specific force (right panel) measurements of EDL muscle from CD and EtOH mice (*n* ≥ 6 per group). (E) Real‐time PCR analysis to evaluate Atrogin‐1 (left panel) and MuRF1 (right panel) mRNA expression levels in gastrocnemius muscle of CD and EtOH mice (*n* ≥ 6 per group). (F) Representative images (left panel) and densitometric analysis (right panel) of western blot for LC3B protein in gastrocnemius muscles of CD and EtOH mice. Densitometric analysis represents the cytosolic protein ratio between the isoforms LC3B‐II/LC3B‐I, indicating the autophagic flux (*n* ≥ 5 per group). Ponceau was used as a loading control. Full‐length western blot images are shown in Figure S1. All data are expressed as mean ± SEM. Data were analysed by Mann–Whitney *U* test. EtOH mice versus CD mice, *p < 0.05 and **p < 0.01.

To verify whether the development of liver disease was associated to sarcopenia, we analysed muscle mass and fibres CSA of the two mouse groups. EtOH mice showed a significant reduction of the weight of different skeletal muscles (Figure 2A), except for soleus, whose mass remained at levels comparable to CD mice. Conversely, no significant difference was observed in body weight (Figure 2A). Histological analysis of the EDL muscle showed a significant decrease of CSA values of myofibers in EtOH mice, compared to those of control mice (Figure 2B). Frequency distribution of CSA further revealed a shift of the median values towards small myofibers size in alcohol‐fed mice (Figure 2C). Functional analysis showed a significant reduction of tetanic force of the muscle of EtOH mice, compared to CD mice (Figure 2D), whereas no difference in specific force was observed (Figure 2D). To investigate the molecular mechanism underpinning loss of muscle mass, we analysed the molecular markers of two proteolytic pathways, the ubiquitin proteasome pathway and the autophagic one. Molecular analysis did not reveal significant modulation in the two E3 ubiquitin ligase expression, namely, Atrogin‐1 and MuRF1 (Figure 2E), whereas the protein LC3‐II/LC3‐I ratio was significantly upregulated in the muscle of EtOH mice, compared to CD mice (Figure 2F), suggesting the involvement of autophagic pathways in muscle loss in ALD [28].

### 3.2 ALD Mice Exhibited Increased Amount of Circulating EVs

**FIGURE 3:**
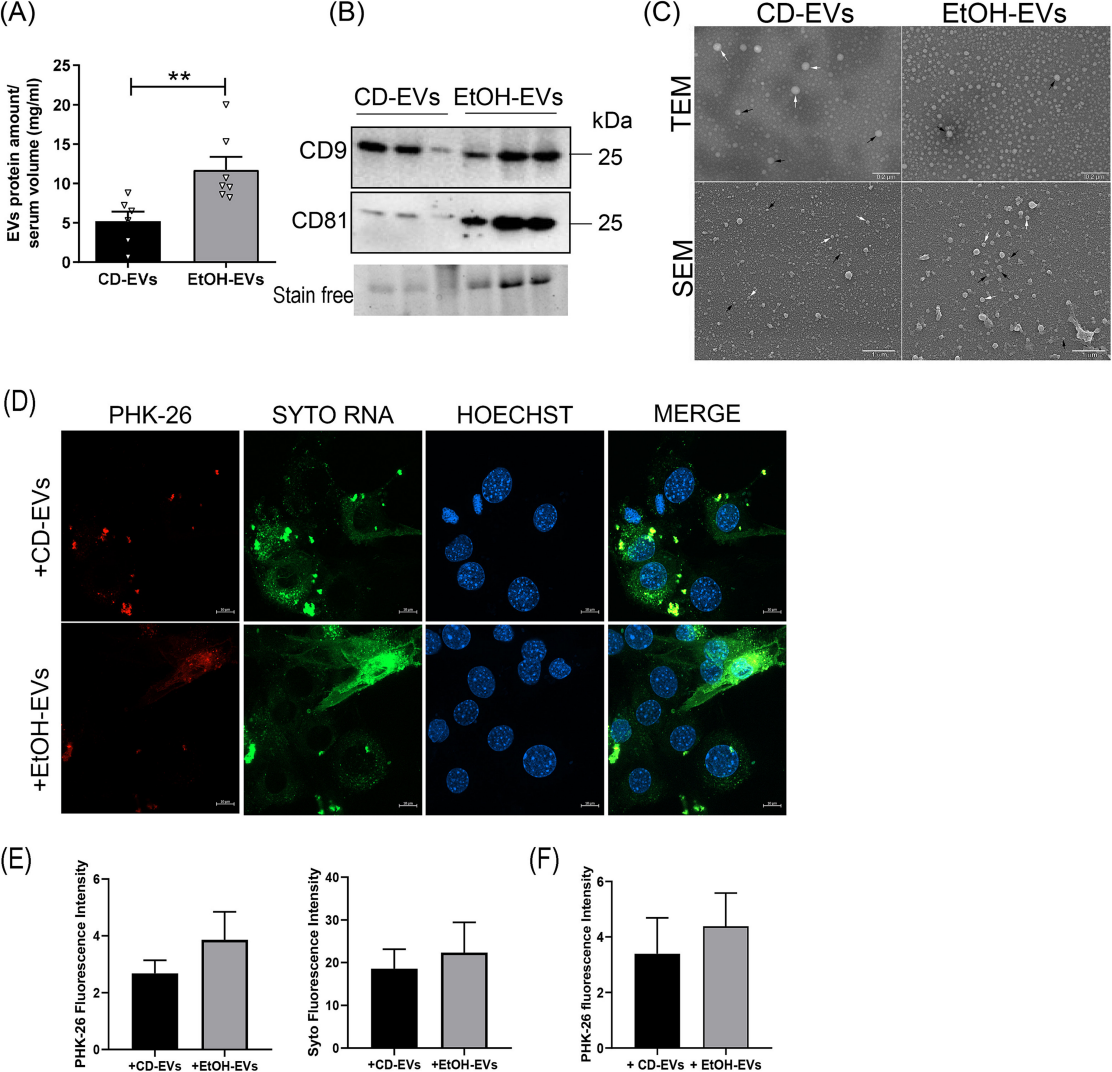
Analysis of EVs isolated from serum of CD and EtOH mice. (A) Determination by Bradford assay of total protein content of EVs isolated from serum of CD and EtOH mice. EV protein amount (mg) is normalized to serum volume (mL) used for EV isolation (*n* ≥ 6 per group). (B) Representative images of western blot for CD9 and CD81 protein of EVs derived from equal serum amount of CD and EtOH mice (*n* ≥ 5 per group). Stain‐free was used as a loading control. Full‐length western blot images are shown in Figure S3. (C) Representative images of transmission electron microscopy (TEM) (upper panels; scale bar: 0.2 μm; black arrows: EVs < 50 nm; white arrows: EVs ≥ 50 nm) and scanning electron microscopy (SEM) (lower panels; scale bar: 1 μm; black arrows: EVs < 100 nm; white arrows: EVs ≥ 100 nm) analyses to test efficiency of EV purification from serum of CD and EtOH mice (*n* ≥ 3 per group). At a lower magnification respect to TEM images, SEM images showed wider frameworks of the EV populations revealing a wider range of vesicle dimensions and the EV spherical morphology. (D) Representative confocal microscopy images of C2C12 cell culture exposed for 3 h to PKH‐26+/SytoRNA+ EVs. Nuclei are stained in blue with Hoechst. SytoRNA positive cells are stained in green (scale bar: 10 μm). (E,F) Fluorescent intensity of PKH‐26 and SytoRNA signals in cell culture after 3 h of exposure to co‐labelled EVs (E, left and right panel, respectively); fluorescent intensity of PKH‐26 signals in cell culture after 24 h of exposure to PKH‐26‐EVs (F) (*n* ≥ 3 per group). All data are expressed as the mean ± SEM. Data are analysed by Mann–Whitney *U* test. EtOH‐EVs versus CD‐EVs, **p < 0.01.

To evaluate whether EtOH feeding could affect the release of EVs, a quantitative analysis of circulating EVs was performed. Protein quantification of EVs isolated from equal volumes of serum from both groups of animals showed higher EV protein amount in EtOH mice (EtOH‐EVs), compared to CD‐ones (CD‐EVs) (Figure 3A), suggesting a greater number of circulating EVs in these mice. These data were partially confirmed by nanoparticle tracking analysis (NTA), revealing a trend of increase in particle concentration of EVs in alcoholic mice (Figure S2). Furthermore, transmission (TEM) and scanning (SEM) electron microscopy analyses showed, despite the similar average particle size between EtOH‐EVs and CD‐EVs (Figure S2) examined by the NTA, an enrichment in exosome‐like vesicles in serum of EtOH mice, compared to that of CD mice (Figure 3C). SEM analysis (Figure 3C, bottom panels) confirmed the vesicular nature of the particles observed by TEM (Figure 3C, top panels). These data were in line with recent evidence highlighting a polarization of vesicle production towards the exosomal component during liver disease [17, 19]. Western blot analysis of the same samples revealed a positive expression of two typical EVs markers, CD9 and CD81, in both CD‐ and EtOH‐EVs (Figure 3B).

Finally, we evaluated the ability of myogenic C2C12 cells to uptake and internalize circulating EVs, as well as to transfer EV cargo into muscle cells. We labelled CD‐EVs and EtOH‐EVs with two fluorescent dyes, namely, the PKH‐26 dye, staining the extracellular vesicle membrane, and the SYTO RNASelect dye, selectively staining the RNA, as important component of the EV cargo; then cell cultures were exposed to PKH‐26+/SYTO+‐EVs for 3 h. Analysis of confocal microscopy showed efficient uptake and cellular internalization of EVs (Figure 3D), although no difference was observed between EtOH‐EVs and CD‐EVs (Figure 3E). Similar efficiency of internalization was observed also at a longer time point, with no differences between the two types of EVs (Figures 3F and S4).

### 3.3 Circulating EVs Isolated From the Mouse Model of ALD Induce Alterations in Muscle

To evaluate whether circulating EVs in liver disease can affect skeletal muscle cells, we exposed muscle C2C12 cell culture to EVs isolated from serum of either EtOH or CD mice.

**FIGURE 4:**
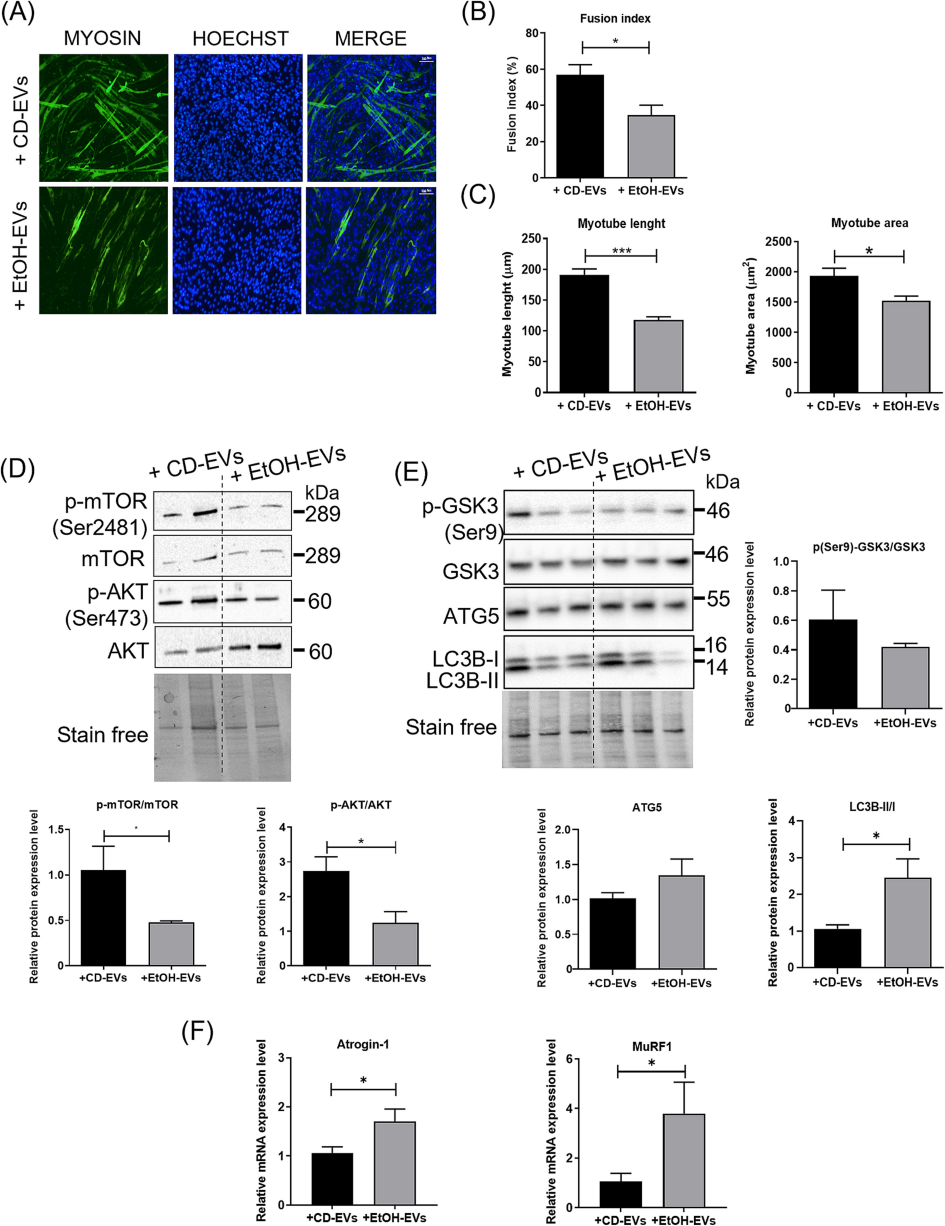
Exposure of C2C12 cell culture to EVs isolated from serum of CD and EtOH mice induced in vitro muscle atrophy. (A) Representative images of immunofluorescence analysis for myosin on C2C12 cell culture on the fifth day in differentiation (DM5) after exposure to serum EVs derived from CD (+CD‐EVs) and EtOH mice (+EtOH‐EVs); EV exposure was performed at time of differentiation (DM0) and after 3 days of differentiation (DM3). (B) Measurement of fusion index and (C) morphometric analysis of myotubes in EV‐treated C2C12 cell culture at DM5. Nuclei and myotubes were examined in five microscopic fields (scale bar: 100 μm) for each group in three independent cultures. (D) Representative images (top panel) and densitometric analysis (bottom panel) of western blot for p‐AKT (Ser473), total AKT, p‐mTOR (Ser2481) and total mTOR proteins in EV‐treated C2C12 cell culture at DM5. Densitometric analysis represents the protein ratio of p‐AKT/AKT and p‐mTOR/mTOR (*n* ≥ 5 for each group in three independent cultures). Full‐length western blot images are shown in Figure S6. (E) Representative images (top panel) and densitometric analysis (right and bottom panels) of western blot for p‐GSK3 (Ser9), total GSK3, ATG5 and LC3B proteins in EV‐treated C2C12 cell culture at DM5. Densitometric analysis represents the protein ratio of p‐GSK3/GSK3 and LC3B‐II/I, whereas ATG5 protein levels were measured normalizing western blot band intensity to stain‐free total lane protein (*n* ≥ 5 for each group in three independent cultures). Stain‐free was used as a loading control. Full‐length western blot images are shown in Figure S7. (F) Real‐time PCR analysis to evaluate Atrogin‐1 and MuRF1 mRNA expression levels in C2C12 cells after exposure to serum CD‐ and EtOH‐EVs at DM5. All data are expressed as mean ± SEM. Data are analysed by Mann–Whitney *U* test. C2C12 cell culture treated with EtOH‐EVs versus culture treated with CD‐EVs; *p < 0.05, ***p < 0.001.

EV treatment of C2C12 cells was performed at the time of their shift into differentiation medium (DM0) and after 3 days (DM3). Then, muscle cultures were analysed at 5 days in differentiation medium (DM5), when fully differentiated myotubes are formed. Interestingly, the analysis of fusion index revealed that circulating EVs isolated from the mouse model of ALD interfered with muscle differentiation impairing the capacity of myoblasts to fuse forming myotubes (Figure 4A,B). Moreover, immunofluorescence analysis at DM5 for the expression of myosin heavy chain (MyHC) showed fewer and smaller myotubes in the cell culture exposed to EVs derived from serum of EtOH‐fed mice, compared to that exposed to EVs from CD mice (Figure 4A,C).

Of note, muscle differentiation resulted also altered in the primary cultures of mouse skeletal muscle cells (MuSCs). As C2C12 cells, primary myoblasts, isolated from skeletal muscles of wild‐type mice, were exposed to serum‐derived EVs at DM0 and DM3 and analysed at DM5. Immunofluorescence analysis for MyHC expression showed, similarly to what observed in the C2C12 cell line, a severe impairment in muscle differentiation induced by EtOH‐EVs, which significantly reduce the fusion index of muscle cells and the size of cultured myotubes, compared to CD‐EVs (Figure S5).

We then evaluated whether the altered myogenic program, induced by EVs derived from serum of EtOH mice, is determined by the inhibition of the canonical pathway promoting muscle differentiation and protein synthesis, namely, AKT kinase and the mammalian target of rapamycin (mTOR) signalling. Western blot analysis revealed a significant decreased phosphorylation of both AKT/mTOR proteins in C2C12 cells exposed to serum‐derived EVs from ALD mice, compared to that exposed to CD‐EVs (Figure 4D). Additionally, we evaluated the activation of protein degradation pathways. Consistent with the downregulated phosphorylation of AKT Ser 473 (Figure 4D), C2C12 cells exposed to EtOH‐EVs also exhibited a decrease in GSK‐3β Ser9 phosphorylation, a negative event that leads to activation of GSK‐3β, which in turn acts as an endogenous inhibitor of protein synthesis and a promoter of protein degradation [S4]. Moreover, C2C12 cell culture exposed to serum EtOH‐EVs exhibited the activation of autophagy pathway, with increased expression levels of ATG5 and of LC3‐II/LC3‐I ratio (Figure 4E), and the induction of the ubiquitin proteasome pathway, with upregulation of Atrogin‐1 and MuRF1 (Figure 4F), compared to culture exposed to CD‐EVs.

### 3.4 Hepatic Extracellular Vesicles Mimic the Effects of Circulating EVs on Skeletal Muscle Cells

We then investigated whether the effects on muscle culture of circulating EVs in ALD could be mainly mediated by liver‐derived EVs [14, 17]. We evaluated whether EVs isolated from liver (hEVs) of EtOH mice affect C2C12 cell culture similarly to circulating EVs isolated from the same animals.

**FIGURE 5:**
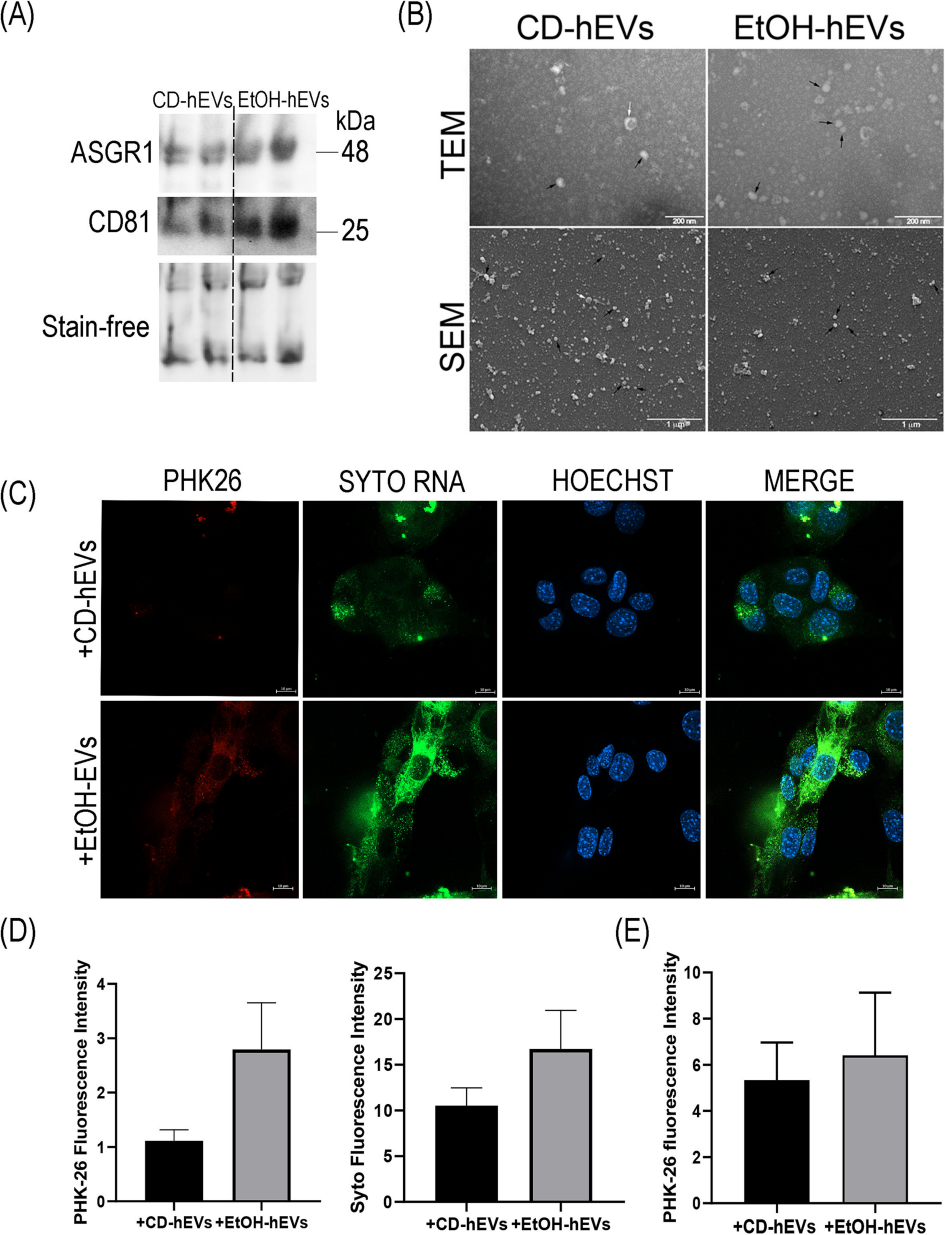
Analysis of hepatic EVs isolated from liver of CD and EtOH mice. (A) Representative images of western blot for ASGR1 and CD81 protein level of hEVs isolated from liver of CD and EtOH mice (*n* ≥ 3 per group). Stain‐free was used as a loading control. Full‐length western blot images are shown in Figure S8. (B) Representative images of transmission electron microscopy (TEM) (upper panels; scale bar: 200 nm; black arrows: EVs ≤ 50 nm; white arrows: EVs > 50 nm) and scanning electron microscopy (SEM) (scale bar: 1 μm; black arrows: EVs ≤ 100 nm; white arrows: EVs > 100 nm) analyses to test efficiency of hEV purification from liver tissue of CD and EtOH mice (*n* ≥ 3 per group). At a lower magnification respect to TEM images, SEM images showed wider frameworks of the EV populations and revealed the EV spherical morphology. (C) Representative confocal microscopy images of C2C12 cell culture exposed for 3 h to PKH‐26+/SytoRNA+‐labelled hepatic EVs. Nuclei are stained in blue with Hoechst. SytoRNA‐positive cells are stained in green (scale bar: 10 μm). (D,E) Fluorescent intensity of PKH‐26 and SytoRNA signals in cell culture after 3 h of exposure to hepatic co‐labelled EVs (E, left and right panel, respectively) (F) (*n* ≥ 3 per group). All data are expressed as the mean ± SEM (*n* ≥ 3). Data were analysed by Mann–Whitney *U* test. EtOH‐hEVs versus CD‐hEVs, *p < 0.05.

Western blot analysis, showing a positive expression of both the typical EVs marker CD81 and the liver‐specific protein ASGR1, confirmed the effective isolation of hepatic extracellular vesicles from liver tissue (Figure 5A). Furthermore, electron microscopy analysis revealed the abundant quantity of vesicles isolated from the liver of animal models (Figure 5B).

Finally, we assessed the ability of C2C12 cells to uptake and internalize liver‐derived EVs. As for circulating EVs, muscle cell cultures were exposed to the PKH‐26+/SYTO RNA+‐labelled hepatic EVs for 3 h and then analysed by confocal microscopy. The results demonstrated an efficient internalization of hepatic EVs within muscle cells, with the transfer of their cargo (Figure 5C). No significant difference was observed between EtOH‐hEVs and CD‐hEVs (Figure 5D). Of note, a similar efficiency of internalization was also observed at 24‐h period of exposure (Figures 5E and S9).

**FIGURE 6:**
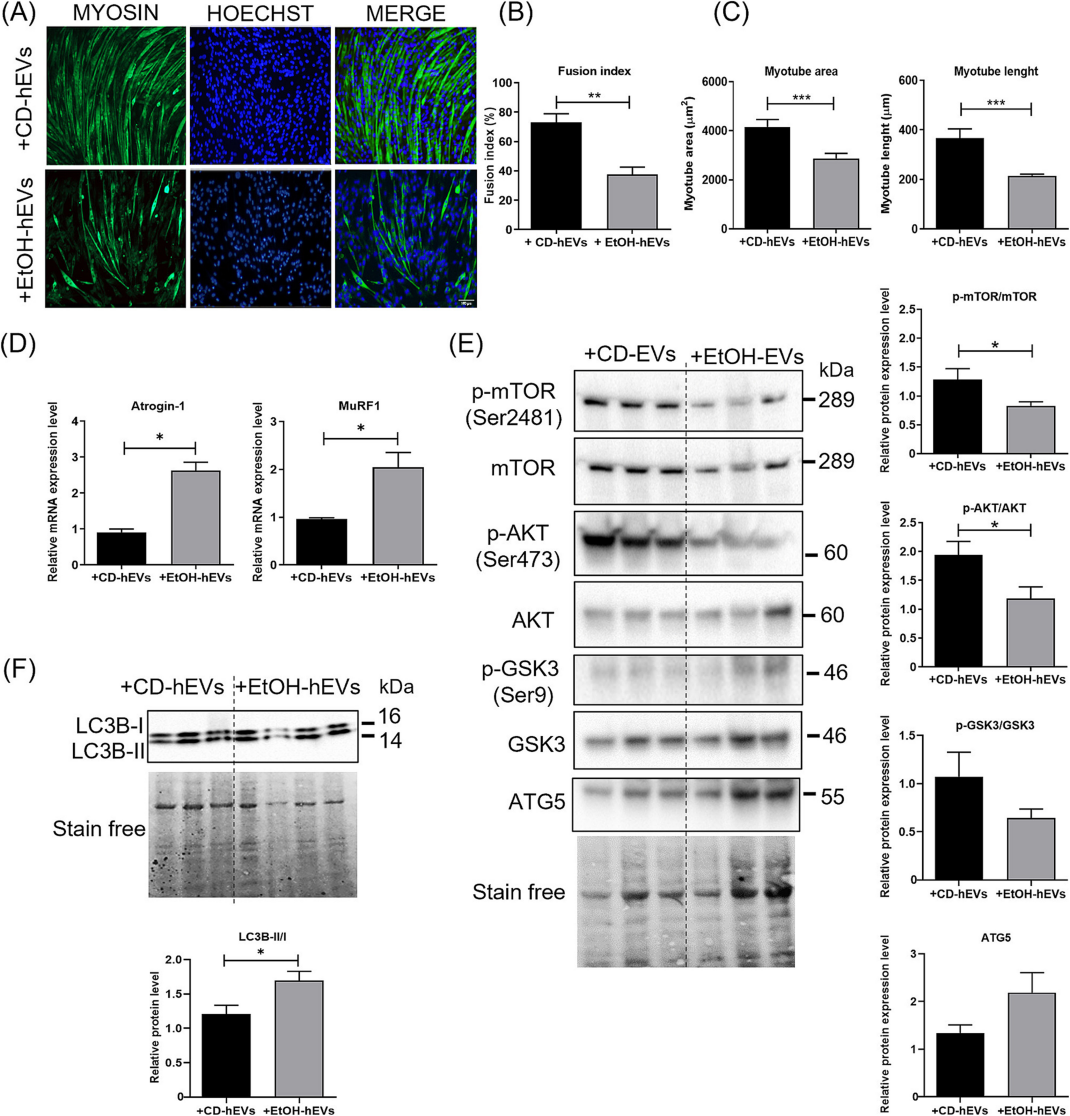
Exposure of C2C12 cell culture to hepatic EVs isolated from CD and EtOH mice induces in vitro muscle atrophy. (A) Representative images of immunofluorescence analysis for myosin on C2C12 cell culture after 5 days of differentiation (DM5) after exposure to hepatic EVs (hEVs) derived from CD mice (+CD‐hEVs) and EtOH mice (+EtOH‐hEVs); hEV exposure was performed at time of differentiation (DM0) and after 3 days of differentiation (DM3) (scale bar: 100 μm). (B) Measurement of Fusion index and (C) morphometric analysis of myotubes in hEV‐treated C2C12 cell culture at DM5. Nuclei and myotubes were examined in five microscopic fields for group in three independent cultures. (D) Real‐time PCR analysis to evaluate Atrogin‐1 (left panel) and MuRF1 (right panel) mRNA expression levels in C2C12 cells exposed to hepatic CD‐EVs and EtOH‐EVs at DM5. (E) Representative images (left panel) and densitometric analysis (right panel) of western blot for LC3B‐II and LC3B‐I proteins in hepatic EV‐treated C2C12 cell culture at DM5. Densitometric analysis represents the protein ratio of LC3B‐II/I (*n* ≥ 5 for each group in three independent cultures). Stain‐free was used as a loading control. Full‐length western blot images are shown in Figure S11. (F) Representative images (left panel) and densitometric analysis (right panels) of western blot for pmTOR (Ser2481), total mTOR, p‐AKT (Ser473), total AKT, p‐GSK3 (Ser9), total GSK3 and ATG5 proteins in hEV‐treated C2C12 cell culture at DM5. Densitometric analysis represents the protein ratio of p‐mTOR/mTOR, p‐AKT/AKT and p‐GSK3/GSK3; ATG5 protein levels were measured normalizing western blot band intensity to stain‐free total lane protein (*n* ≥ 5 for each group in three independent cultures). Stain‐free was used as a loading control. Full‐length western blot images are shown in Figure S12. All data are expressed as mean ± SEM. Data are analysed by Mann–Whitney *U* test. C2C12 cell culture treated with EtOH‐hEVs versus culture treated with CD‐hEVs, *p < 0.05, **p < 0.01, ***p < 0.001.

Furthermore, hEVs from EtOH mice were able to induce a significant reduction of muscle differentiation of C2C12 culture, as revealed by decreased values of fusion index (Figure 6A,B) and myotube size (Figure 6A,C), compared to hEVs from CD mice. Similar effects were observed also in primary culture of MuSCs (Figure S10). Moreover, EtOH‐hEVs, similarly to circulating ones, and in comparison with CD‐hEVs, induced upregulation of markers of both ubiquitin proteasome (Figure 6D) and autophagy systems (Figure 6E,F) [28] and downregulation of markers of protein synthesis (Figure 6E).

### 3.5 Analysis of miRNA Expression in EVs Isolated From Serum and Liver of the Mouse Model of ALD

**FIGURE 7:**
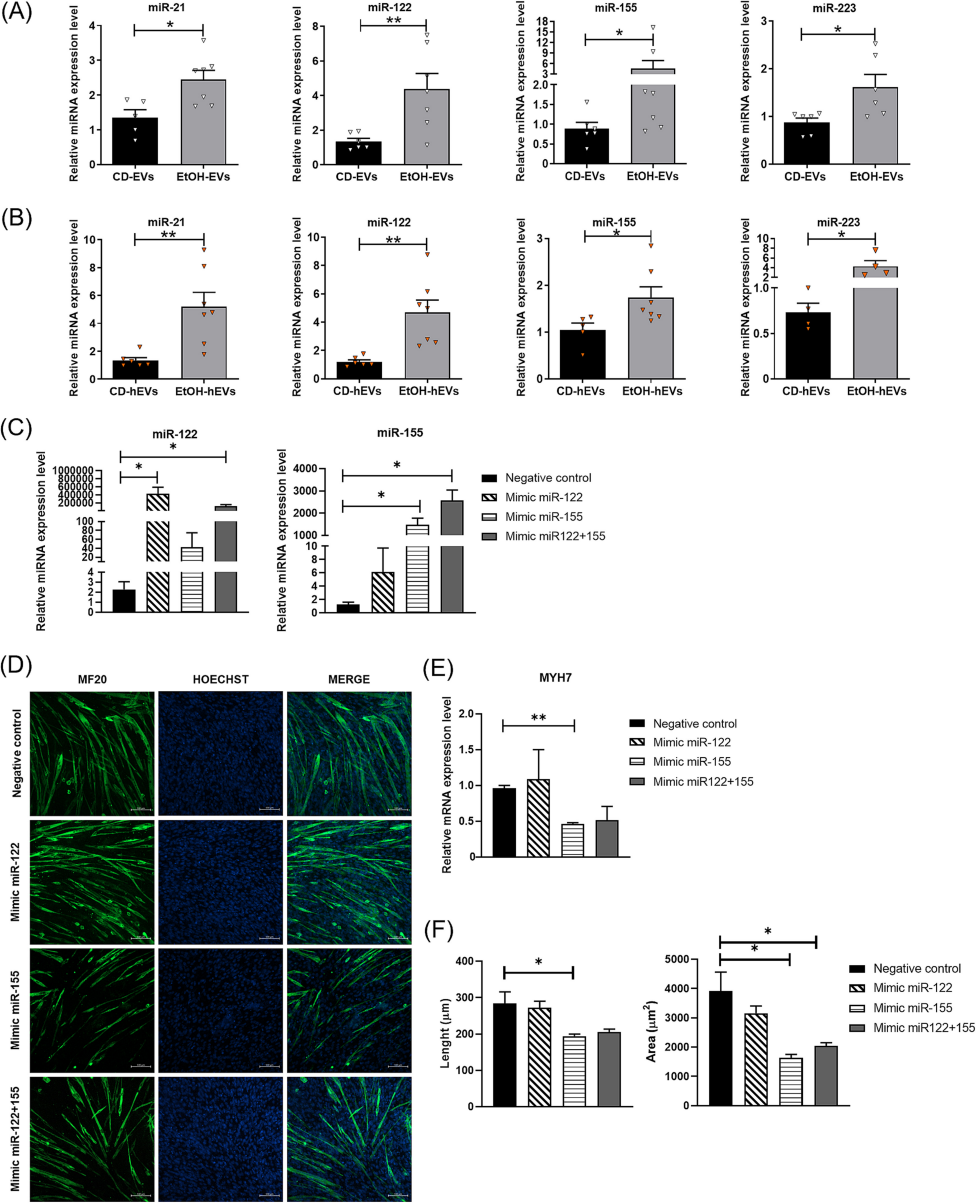
Similar expression pattern of miRNAs between circulating, hepatic EVs and skeletal muscle tissue.(A‐B) Real‐time PCR analysis to evaluate miR‐21, miR‐122, miR‐155 and miR‐223 expression levels in EVs isolated from serum (A) and liver tissue (B) of CD and EtOH mice (*n* ≥ 5). All data are expressed as the mean ± SEM. Data were analysed by Mann–Whitney *U* test. EtOH‐EVs versus CD‐EVs, EtOH‐hEVs versus CD‐hEVs EtOH mice versus CD mice, *p < 0.05, **p < 0.01. (C) Real‐time PCR analysis to evaluate miR‐122 (left panel) and miR‐155 (right panel) expression levels in C2C12 cells 72 h after mimic administration (*n* ≥ 3). (D) Representative confocal microscopy images of C2C12 cell culture immunostained with MF20 after 72 h of exposure to miR‐112 or miR‐155 mimics or both. Mimics transfection was performed at the time of the shift in differentiation medium (DM0). Nuclei and myotubes were examined in five microscopic fields for each group in three independent cultures. (E) Morphometric analysis of myotubes in C2C12 cells after 72 h of miRNA mimics transfection. (E) Real‐time PCR analysis to evaluate Myh7 expression levels after 72 h of miRNA mimics transfection (*n* ≥ 3). All data are expressed as the mean ± SEM. Data were analysed by one‐way ANOVA. Mimic versus negative control, *p < 0.05.

Based on the critical role of EV‐miRNAs in liver diseases [19] and on their ability to regulate tissue homeostasis in an autocrine/paracrine and endocrine fashion, we analysed the miRNAs cargo of circulating EVs in the mouse model of ALD, examining microRNAs specifically involved in liver disease and in muscle atrophy. We evaluated the expression levels of miR‐21, miR‐155 and miR‐223, besides those of the hepato‐specific miR‐122 [18, 29]. RT‐PCR analyses showed significant upregulation of all miRNAs in serum EVs isolated from alcoholic mice, compared to those from CD animals (Figure 7A). To evaluate the hypothesis that the circulating EVs could deliver their cargo to skeletal muscle, we also analysed the expression levels of these microRNAs in muscle tissue of EtOH and CD mice. Notably, the higher expression levels of all examined miRNAs also in muscle (Figure S13A,B) suggested that they are carried by EVs to skeletal muscle, likely contributing to the sarcopenic process.

The elevated expression of the liver‐specific miR‐122, both in circulating EVs (Figure 7A) and in muscle tissue (Figure S13B) of EtOH mice, could indicate a hepatic origin of serum EVs and of their delivery to muscle tissue.

However, to potentially exclude a transcriptional induction of miR‐122 in skeletal muscle of sarcopenic ALD mice, we compared its muscle expression in EtOH mice with that in 24‐month‐old mice, which displayed age‐related sarcopenia (Figure S13B). The lack of miR‐122 modulation in older mice, contrarily to alcoholic ones, supports the hypothesis of its EV‐mediated delivery to muscle tissue in ALD condition.

To further investigate the liver tissue‐derivation, we analysed the expression of the same microRNAs also in EVs isolated from liver of EtOH and CD mice (Figure 7B). The similar expression pattern of these miRNAs between the circulating and the hepatic EVs, with a significant upregulation of them in EtOH mice compared to CD animals, additionally suggested that liver in pathological condition could be an important source of circulating EVs.

To evaluate the direct and specific effects of these miRNAs on muscle cells, we analysed their overexpression in muscle cells, transfecting C2C12 cells by the corresponding miRNA mimics. We examined the effects of miR‐122 overexpression, as it is the most represented hepatic microRNA, and of miR‐155, as it was more upregulated in circulating and in skeletal muscle of ALD mice (Figures 7A and S13). The analysis conducted after 72 h from mimic administration, corresponding to the third day in differentiation medium (DM3), resulted in a remarkable upregulation of mir‐122 and miR‐155 (Figure 7C). Analysis of C2C12 cells, exposed to miR‐155 mimic, showed at DM3 a significant reduction of myotube size (Figure 7D,F) and a downregulation of the myosin heavy chain (Myh7) (Figure 7E). Conversely, overexpression of miR‐122 did not induce significant alteration in muscle cell culture, whereas administration of both mimics led to a reduction in myotube size (Figure 7D,F) and in Myh7 expression (Figure 7E).

Of note, overexpression of miR‐122 also induced higher expression of endogenous mir‐155, compared to the negative control, whereas forced expression of miR‐155 did not modulate miR‐122 expression, suggesting that miR‐122 could be not transcriptionally induced in muscle.

### 3.6 Circulating EVs Isolated From Cirrhotic Patients Impinge Muscle Differentiation

To prove that circulating EVs released from damaged liver are critical to promoting sarcopenia in human liver disease, we investigated the effects of EVs isolated from serum of patients with alcohol‐related cirrhosis (CLD‐EVs) on human muscle cell culture, comparing them with those of EVs from healthy individuals (H‐EVs).

**FIGURE 8:**
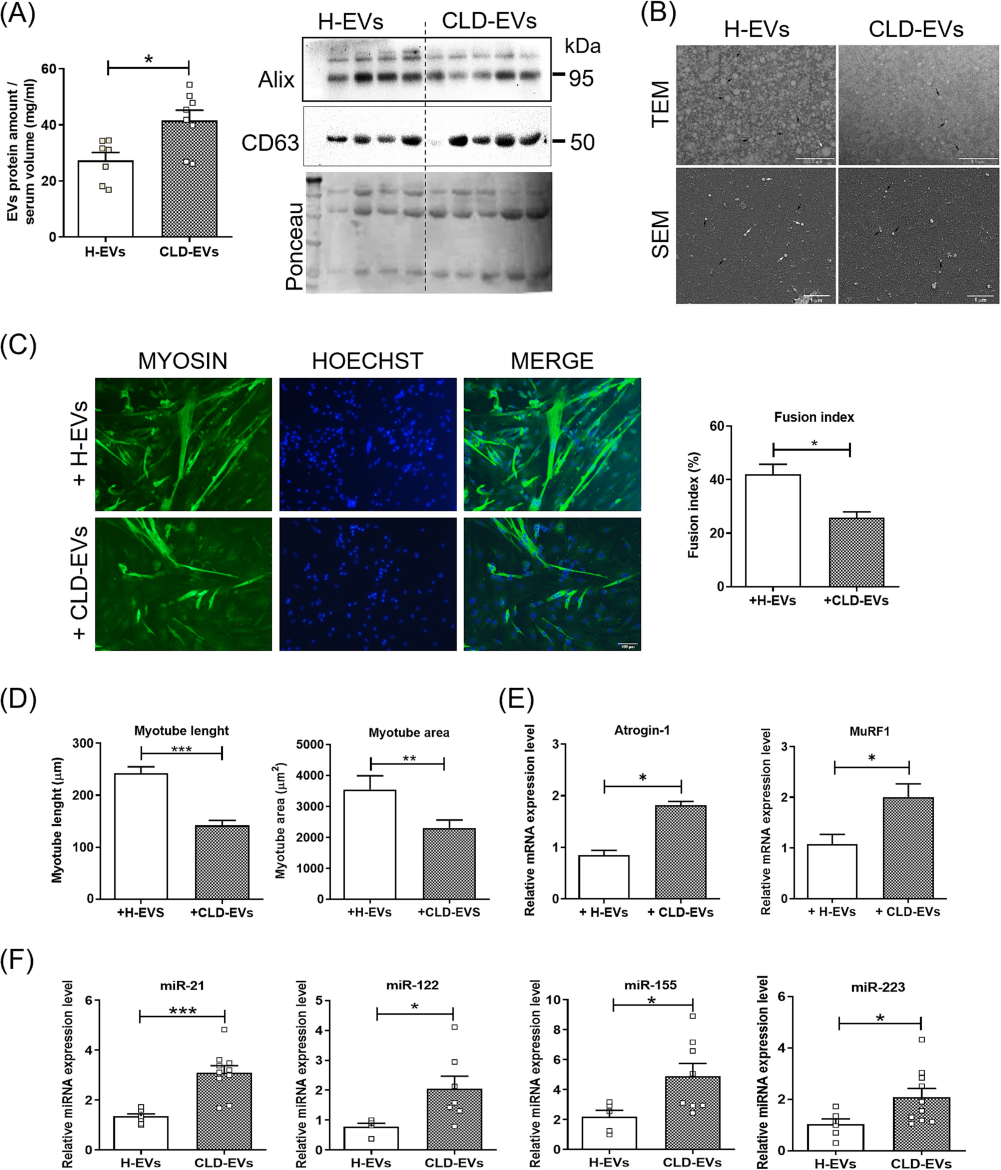
Analysis of EVs isolated from serum samples of healthy individuals (H‐EVs) and cirrhotic patients (CLD‐EVs) and evaluation of their effects on muscle cells. (A) Determination by Bradford assay of total protein content of EVs isolated from serum of healthy donors and CLD patients (A, left panel). EV protein amount (mg) is normalized to serum volume (mL) used for EV isolation (*n* ≥ 7 per group). Representative images of western blot for Alix and CD63 protein of EVs derived from equal serum amount of healthy donors and CLD patients (*n* ≥ 4 per group) (A, right panel). (B) Representative images of transmission electron microscopy (TEM) (upper panels; scale bar: 0.5 μm; black arrows: EVs ≤ 50 nm; white arrows: EVs > 50 nm) and scanning electron microscopy (SEM) (lower panels; scale bar: 1 μm; black arrows: EVs < 100 nm; white arrows: EVs ≥ 100 nm) analyses to test efficiency of EV purification from serum of healthy donors and CLD patients (*n* ≥ 3 per group). At a lower magnification respect to TEM images, SEM images showed wider frameworks of the EV populations and revealed the EV spherical morphology. (C) Representative images of immunofluorescence analysis for myosin on primary culture of human skeletal muscle cells on the fifth day in differentiation medium (DM5) after exposure to EVs derived from serum of H and CLD patients (C, left panel) (scale bar: 100 μm). EV exposure was performed at time of differentiation (DM0) and after 3 days of differentiation (DM3). Measurement of fusion index of myotubes in EV‐treated human skeletal muscle cells at DM5 (C, right panel). (D) Morphometric analysis of myotubes in EV‐treated human skeletal muscle cells at DM5. Nuclei and myotubes were examined in five microscopic fields for each group in three independent cultures. (E) Real‐time PCR analysis to evaluate Atrogin‐1 (left panel) and MuRF1 (right panel) mRNA expression levels in human primary skeletal muscle cells after exposure to HD‐ and CLD‐EVs at DM5. (F) Real‐time PCR analysis to evaluate miR‐21, miR‐122, miR‐155 and miR‐223 expression levels in EVs isolated from serum CLD and healthy individuals (*n* ≥ 5 per group). All data are expressed as mean ± SEM. Statistical analysis: non‐parametric test, Mann Whitney *U* test, *p < 0.05 and **p < 0.01; * represents the significance with the healthy subjects.

Protein quantification of EVs isolated from equal volumes of serum from patients and control group revealed, similarly to animal models, higher protein amount in cirrhotic patients (Figure 8A, left panel). However, this increase was not correlated with a greater number of vesicles, as assessed by NTA, but, unlike in EtOH mice, to an increased average size of the particles (Figure S14).

Moreover, western blot analysis of equal protein amount of CLD‐EVs and H‐EVs showed a positive expression of two typical EVs markers, such as Alix and CD63 (Figure 8A, right panel). As observed in circulating EVs isolated from mice, SEM and TEM analyses showed, based on size, an enrichment in exosomes in serum samples of CLD patients compared to H‐EVs (Figure 8B). Furthermore, we demonstrated that human primary muscle cells, as MuSCs and C2C12 cells, were able to uptake circulating EVs of both patients and healthy subjects (Figure S16).

Treatment of human muscle cell cultures with serum‐derived EVs from cirrhotic patients at DM0 and DM3 resulted in a decreased fusion of myoblasts (Figure 8C) and a reduction in myotube size (Figure 8D). As for C2C12 cells treated with EVs from ALD mice, this was associated to a downregulation of the AKT/mTOR pathway, accompanied by a reduced phosphorylation of GSK‐3β(Ser9) (Figure S17) and by a significant upregulation of Atrogin‐1 and MuRF1 (Figure 8E), compared to culture exposed to EVs from healthy subjects. However, no significant differences in the expression of autophagy markers were observed between human muscle cultures exposed to CLD‐EVs and those exposed to HD‐EVs (Figure S17).

Given the similar effects of circulating EVs on muscle cells, between the mouse model of ALD and the alcoholic cirrhotic patients, we investigated whether human CLD‐EVs carry a miRNA cargo comparable to that of murine EtOH‐EVs. We analysed EVs isolated from serum of patients and healthy subjects for the expression of the same miRNAs examined in murine circulating EVs. As in EtOH mice, qPCR analyses showed upregulation of miR‐21, miR‐122, miR‐155 and miR‐223 in the circulating EVs of alcoholic patients (Figure 8F), compared in those from healthy individuals. Of note, the significantly increased expression of the liver‐specific miR‐122 in these vesicles (Figure 8F) suggested, also in human liver disease, a hepatic origin of circulating EVs.

## 4 Discussion

Sarcopenia is a condition highly prevalent in patients with ALD, contributing to adverse clinical outcomes and to elevated risk of mortality [3]. However, the physiopathologic mediators of the liver–muscle interplay remains elusive. As cellular response to liver damage [16, 19], hepatic cells can release into circulation an increased number of EVs [13], which have recently emerged as novel mediators of the intercellular communication [30, 31, 32, 33]. Consequently, we aimed to evaluate the role of circulating EVs in sarcopenia associated to liver disease.

To this, we analysed the effects of serum EVs on muscle cell culture in a mouse model of ALD, specifically generated for this study, and in human, by isolating EVs from serum of patients with alcohol‐related cirrhosis.

We demonstrated the efficient uptake and internalization of murine and human circulating EVs, as well as of murine hepatic EVs, in muscle cells. Strikingly, these EVs were able to impinge the myogenic program and the protein turnover regulation in both cell line and muscle primary cultures, interfering with muscle differentiation. At molecular level, circulating EVs induced a downregulation of PI3K/AKT/mTOR signalling pathway, which is crucial for protein synthesis and muscle differentiation [7], and an upregulation of protein degradation pathways, namely, autophagy and ubiquitin proteasome pathways, with a dominant role of the last in human culture.

The study highlights a direct effect of hepatic EVs on skeletal muscle cells, suggesting that circulating EVs mediating sarcopenia in chronic liver disease likely originate from the liver. In this context, the generation of mouse model of ALD, which we have firstly characterized to assess its suitability in our study, played a crucial role in exploring the role of EVs in sarcopenia associated with liver disease.

Our data revealed that the effects of EVs on muscle cells could be mediated by a specific cargo of miRNAs, which has been implicated in pathogenesis of acute and chronic liver diseases [18, 19] and in regulating muscle development and homeostasis [32].

We focused on microRNAs reported to be involved in ALD and associated to muscle atrophy, such as miR‐21, miR‐155, miR‐223 and miR‐122 [18, 32].

The elevated expression levels of these microRNAs in circulating and hepatic EVs from the ALD mouse model and cirrhotic patients, compared to healthy controls, are related to their crucial role in alcoholic liver pathogenesis.

MiR‐21, which is found upregulated in the liver of patients with alcoholic hepatitis, is a putative mediator of hepatic damage, regulating tissue repair during alcohol exposure [34]. MiR‐155, whose expression is induced by ethanol, promotes the release of EVs from liver cells [18], targeting multiple genes of autolysosomal degradation, whereas miR‐223 increases in serum and in neutrophils of alcoholics and mouse models of ALD, where it inhibits the interleukin‐6–p47phox–ROS pathway, limiting cell infiltration and protecting against alcohol‐induced liver injury [35]. MiR‐122 is a hepato‐specific miRNA, which increases in circulation in almost all liver diseases [19, 36]. Additionally, miR‐122 is enriched in EVs produced in hepatocytes after alcohol exposure, mediating cellular communication between hepatocytes and monocytes and thus contributing to the pathogenic progression of alcoholic hepatitis [19].

These miRNAs also play a role in muscle homeostatic impairment. MiR‐21 regulates muscle development and mass [37]; it is associated to sarcopenia and to a disease‐related decline of muscle regeneration [38]. MiR‐155 directly targets MEF2A, modulating satellite cell activity, decreasing myoblast differentiation and inducing muscle cell atrophy [39]. MiR‐223 induces a reduction in protein synthesis by targeting IGF‐2 and inhibiting the anabolic pathway of PI3K/AKT/mTOR.

In our study, the elevated expression of the liver‐specific miR‐122 in circulating EVs of ALD mice and cirrhotic patients suggests the hepatic origin of serum EVs or, to some degree, an enriched fraction of liver‐derived EVs in circulation.

To elucidate the specific impact of these miRNAs on muscle cells, we overexpressed selected miRNAs in muscle cells. Of note, overexpression of miR‐155 led to a significant reduction in myotube size and downregulation of myosin. In contrast, the overexpression of miR‐122 itself did not significantly alter the muscle cell culture at the early stage of differentiation, whereas administration of both mimics, namely, miR‐155 and miR‐122, negatively impacted muscle differentiation. The lack of the effect of miR‐122 mimic does not rule out its potential impact at later stages of muscle differentiation. Previous studies have indicated that miR‐122 promotes skeletal muscle proteolysis when transfected into myotubes, implying that its effects might become more evident at later differentiation stages [40]. Further study is needed to explore the temporal effects of miR‐122 and to define the underlying mechanisms of miRNA interactions in muscle cells.

Although we did not directly assess the effects of the overexpression of the other microRNAs, namely, miR‐223 and miR‐21, different studies support the role of these miRNAs in mediating muscle atrophy in cell culture. Transfection of miR‐223 mimic inhibited myoblast proliferation [S5], whereas miR‐21 overexpression negatively regulated myoblast viability and differentiation [38]. It is worth to consider that muscle differentiation, homeostasis and function are regulated by different signals and molecules and that a single factor can potentially modulate or impact only part of this complex program. It is plausible that the different EVs cargo molecules act synergistically or in a combinatorial fashion to modulate the complexity of muscle homeostasis and to promote muscle alterations.

In conclusion, our results demonstrated that the negative effect of circulating EVs from ALD mice and cirrhotic patients in muscle cell culture could be mediated by their miRNA cargo, targeting molecular factors involved in muscle homeostasis and differentiation. The higher expression of these microRNAs also in the skeletal muscle of the mouse model of ALD compared to CD mice indicated the ability of circulating EVs to deliver this miRNA cargo to the distal muscle tissue contributing to the sarcopenic process. Moreover, the similar expression pattern of all examined miRNAs between the circulating and hepatic EVs further confirms that, in pathological condition, the liver is an important source of circulating EVs, which induce alteration in the myogenic program and sarcopenia.

## Author Contributions

Conceptualization: A.M. Methodology: L.B., C.P. and B.P. Investigation: L.B., C.P., C.B., M.C., F.I., C.N., F.F., B.P. and G.B. Patient recruitment and sample collection: S.D.C., L.L. and G.C. Visualization: L.B., C.P., C.B., M.C., F.I., F.F. and G.D. Funding acquisition: A.M. and M.M. Project administration: A.M. Supervision: A.M. Writing–original draft: L.B. and C.P. Writing–review and editing: A.M. and M.M.

## Ethics Statement

The study was conducted according to the guidelines of the Declaration of Helsinki and approved by the Institutional Review Board of the animal facilities of DIEM and National Institute of Health‐Italy (n° 609/2015‐PR; n° 864/2020‐PR). All subjects gave informed consent for the procedure detailed in the paragraph of material and methods. Blood sampling was performed according to a protocol approved by the Ethical Committee of Sapienza University of Rome (Prot 0057/2023).

## Consent

The authors have nothing to report.

## Conflicts of Interest

The authors declare no conflicts of interest.

## Supporting information


**Figure S1** Full‐length western blot images for Figure 2F.
**Figure S2.** Characterization of isolated extracellular vesicles from serum of CD and EtOH mice by nanoparticle tracking analysis.
**Figure S3.** Full‐length western blot images for Figure 3B.
**Figure S4.** Representative confocal microscopy images of C2C12 cell culture exposed for 24 h with PKH‐26‐labelled serum EVs.
**Figure S5.** Analysis of primary culture of mouse skeletal muscle cells (MuSCs) exposed to EVs isolated from serum of CD and EtOH mice.
**Figure S6.** Full‐length western blot images for Figure 4D.
**Figure S7.** Full‐length western blot images for Figure 4E.
**Figure S8.** Full‐length western blot images for Figure 5A.
**Figure S9.** Representative confocal microscopy images of C2C12 cell culture exposed for 24 h to PKH26‐labelled hepatic EVs.
**Figure S10.** Analysis of primary culture of mouse skeletal muscle cells (MuSCs) exposed to hepatic Evs (hEVs) isolated from liver of CD and Et OH mice.
**Figure S11.** Full‐length western blot images for Figure 6E.
**Figure S12.** Full‐length western blot images for Figure 6F.
**Figure S13.** miRNA expression in skeletal muscle.
**Figure S14.** Characterization of isolated extracellular vesicles from serum of healthy subjects and cirrhotic patients (CLD) by nanoparticle tracking analysis.
**Figure S15.** Full‐length western blot images for Figure 8A (right panel).
**Figure S16.** Representative confocal microscopy images of human muscle cells exposed 24 h with PKH26‐labelled EVs.
**Figure S17.** In vitro treatment of human muscle cell culture with circulating EVs isolated from serum samples of healthy individuals (H‐EVs) and cirrhotic patients (CLD‐EVs) induced muscle atrophy.
**Figure S18.** Full‐length western blot images for Figure S17 (left panel).

## Data Availability

The datasets used and/or analysed during the current study are available from the corresponding authors on reasonable request.
