# Supplementary material for: Circulating Extracellular Vesicles in Alcoholic Liver Disease Affect Skeletal Muscle Homeostasis and Differentiation
Source: J Cachexia Sarcopenia Muscle. 2025 Feb 7;16(1):e13675. doi: 10.1002/jcsm.13675 (PMC11806195; doi:10.1002/jcsm.13675)
Supplement: Supplementary file 1 — Figure S1 Full‐length western blot images for Figure 2F. Figure S2. Characterization of isolated extracellular vesicles from serum of CD and EtOH mice by nanoparticle tracking analysis. Figure S3. Full‐length western blot images for Figure 3B. Figure S4. Representative confocal microscopy images of C2C12 cell culture exposed for 24 h with PKH‐26‐labelled serum EVs. Figure S5. Analysis of primary culture of mouse skeletal muscle cells (MuSCs) exposed to EVs isolated from serum of CD and EtOH mice. Figure S6. Full‐length western blot images for Figure 4D. Figure S7. Full‐length western blot images for Figure 4E. Figure S8. Full‐length western blot images for Figure 5A. Figure S9. Representative confocal microscopy images of C2C12 cell culture exposed for 24 h to PKH26‐labelled hepatic EVs. Figure S10. Analysis of primary culture of mouse skeletal muscle cells (MuSCs) exposed to hepatic Evs (hEVs) isolated from liver of CD and Et OH mice. Figure S11. Full‐length western blot images for Figure 6E. Figure S12. Full‐length western blot images for Figure 6F. Figure S13. miRNA expression in skeletal muscle. Figure S14. Characterization of isolated extracellular vesicles from serum of healthy subjects and cirrhotic patients (CLD) by nanoparticle tracking analysis. Figure S15. Full‐length western blot images for Figure 8A (right panel). Figure S16. Representative confocal microscopy images of human muscle cells exposed 24 h with PKH26‐labelled EVs. Figure S17. In vitro treatment of human muscle cell culture with circulating EVs isolated from serum samples of healthy individuals (H‐EVs) and cirrhotic patients (CLD‐EVs) induced muscle atrophy. Figure S18. Full‐length western blot images for Figure S17 (left panel). [file JCSM-16-e13675-s001.pdf]

Supporting information

Circulating extracellular vesicles in alcoholic liver disease affect skeletal muscle homeostasis and differentiation

Laura Barberi[1*], Cristiana Porcu[1*], Caterina Boccia[1], Marianna Cosentino[1], Carmine Nicoletti[1], Barbara Peruzzi[2], Francesca Iosi[3], Flavia Forconi[1], Giulia Bagnato[1,2], Gabriella Dobrowolny[1], Simone Di Cola[4], Lucia Lapenna[4], Gianluca Cera[5], Manuela Merli[4, #], Antonio Musarò[1,6, #]

*These authors contributed equally to this work.

[1] DAHFMO-Unit of Histology and Medical Embryology, Sapienza University of Rome, Laboratory affiliated to Istituto Pasteur Italia – Fondazione Cenci Bolognetti; Rome 00161, Italy.

[2] Bone Pathophysiology Research Unit, Bambino Gesù Children's Hospital, IRCCS, Rome, Italy.

[3] Core Facilities, Microscopy Area, Istituto Superiore di Sanità, Rome 00184, Italy.

[4] Department of Translational and Precision Medicine, Sapienza University of Rome; Rome 00184, Italy

[5] Department of Orthopaedics and Traumatology, Policlinico Umberto I, Rome 00184, Italy.

[6] Scuola Superiore di Studi Avanzati Sapienza (SSAS), Sapienza University of Rome, 00185 Rome, Italy

#Address correspondence; E-mail: antonio.musaro@uniroma1.it; manuela.merli@uniroma1.it

Contents

Supplementary Methods

- Extracellular vesicles isolation from serum and liver samples

- Electron Microscopy

- Primary culture of mouse skeletal muscle cells and EVs treatment

- Primary human myoblasts and treatment

- Extracellular vesicles staining and EVs uptake

- Nanoparticle Tracking Analysis (NTA) of EVs

- miRNAs cell transfection

- Protein extraction and Western Blot

- Histological analyses

- Immunofluorescence analysis on liver tissue and muscle cells

Supplementary Figures

-Figure S1. Full-length Western blot images for Figure 2F.

-Figure S2. Characterization of isolated extracellular vesicles from serum of CD and EtOH-mice by Nanoparticle tracking analysis.

-Figure S3. Full-length Western blot images for Figure 3B.

-Figure S4. Representative confocal microscopy images of C2C12 cell culture exposed for 24h with PKH-26- labeled serum EVs.

-Figure S5. Analysis of primary culture of mouse skeletal muscle cells (MuSCs) exposed to EVs isolated from serum of CD- and EtOH-mice.

-Figure S6. Full-length Western blot images for Figure 4D.

-Figure S7. Full-length Western blot images for Figure 4E.

-Figure S8. Full-length Western blot images for Figure 5A.

-Figure S9. Representative confocal microscopy images of C2C12 cell culture exposed for 24h to PKH26- labeled hepatic EVs.

-Figure S10. Analysis of primary culture of mouse skeletal muscle cells (MuSCs) exposed to hepatic Evs (hEVs) isolated from liver of CD- and Et-OH mice.

-Figure S11. Full-length Western blot images for Figure 6E.

-Figure S12. Full-length Western blot images for Figure 6F.

-Figure S13. miRNAs expression in skeletal muscle.

-Figure S14. Characterization of isolated extracellular vesicles from serum of healthy subjects and cirrhotic patients (CLD) by Nanoparticle tracking analysis.

-Figure S15. Full-length Western blot images for Figure 8A (right panel).

-Figure S16. Representative confocal microscopy images of human muscle cells exposed 24h with PKH26- labeled EVs.

-Figure S17. *In vitro* treatment of human muscle cell culture with circulating EVs isolated from serum samples of healthy individuals (H-EVs) and cirrhotic patients (CLD-EVs) induced muscle atrophy.

-Figure S18. Full-length Western blot images for Figure S17 (left panel).

-Additional references

Supplementary Methods

-Extracellular vesicles isolation from serum and liver samples

Serum extracellular vesicles isolation

EVs were isolated from mice and human serum samples using ExoQuick™ Exosome Precipitation Solution (System Biosciences Inc., CA, USA), following the manufacturer's recommendations. The EVs pellet was resuspended in PBS for subsequent analysis or in differentiation medium for the treatment of cell culture. EVs amount was determined by quantification of EVs total protein with Bradford Protein Assay (Bio-Rad, Inc). EVs protein amount (mg) was normalized to serum volume (ml) used for EVs isolation.

Interstitial EVs isolation from liver of mice

The isolation of interstitial EVs from liver was obtained as described by Matejovic et al.,[21]. Left ventricle perfusion was performed using 10 ml PBS /0.5 mM EGTA (2,5mL/min for 4min) followed by a second perfusion with 10 ml PBS containing 0.5% BSA. The whole liver (~ 1 g) was digested in RPMI 1640 medium containing 2 mg/mL collagenase D and 40 U/mL DNase I. Tissue suspension were subjected to differential centrifugation at 4 °C (300 g for 10 min; 2000 g for 20 min and 10 000 g for 45 min) and filtered through a 0.22-μm membrane. Then, supernatant was ultra-centrifuged at 120000 g over night at 4 °C (SW41 Rotor, Optima LX-80; Beckman Coulter). The pellet was resuspended in PBS and ultracentrifuged at 120 000 g for 3h at 4 °C. The interstitial EVs pellet was resuspended in 100 μl PBS and stored at -80°C for further analysis. Amount of hepatic EVs was determined by quantification of EVs total protein with Bradford Protein Assay (Bio-Rad, Inc); the hEVs protein amount (μg) was normalized to weight (mg) of liver tissue sample.

- Electron Microscopy

Serum and liver-derived EVs were resuspended in PBS and adsorbed onto formvar-carbon-coated grids [21]. Ammonium molybdate (4%, pH 6.4) was added as a contrasting solution and then absorbed with filter paper. Samples were air-dried and observed using a PHILIPS EM208S TEM (ThermoFisher). Additionally, PBS-resuspended EVs were allowed to adhere to polylysine-treated round glass coverslips for 1 hour. Afterward, they were fixed with 2.5% glutaraldehyde and post-fixed with 1% OsO_4, both in sodium cacodylate buffer (0.1M) for 1 hour at room temperature (RT). Samples were dehydrated through a graded series of ethanol solutions (ranging from 30% to 100%) and then in absolute hexamethyldisilazane (HMDS) for 1 hour. After complete removal of HMDS, the samples were left to dry for 2 hours. Glass coverslips were mounted on stubs, lightly gold-sputtered, and analyzed using a field emission SEM Quanta INSPECT F (FEI- ThermoFisher).

-Primary culture of mouse skeletal muscle cells and EVs treatment

Satellite cell populations (MuSCs) from wild-type mice C57BL/6J were isolated using the MACS® Separation Principle (Miltenyi Biotec s.r.l). Skeletal muscle was digested using the Skeletal Muscle Dissociation Kit and dissociated cells were treated with the Satellite Cell Isolation Kit (Miltenyi Biotec s.r.l.) [22]. Unlabeled MuSCs were collected, purified using Anti-Integrin -7 MicroBeads, and then plated on collagen-I coated dishes. MuSCs were cultured in growth medium (GM), containing DMEM supplemented with 20% horse serum (Sigma Aldrich) and 3% chick embryo extract, and changed to differentiation medium (DM), containing DMEM supplemented with 2% horse serum (Sigma Aldrich), after 3 days in culture. Cells were treated, with 30 μg/ml of EVs isolated from serum and liver of CD- and EtOH-mice at the time of their shift into differentiation medium (DM0) and after 3 days (DM3) and were analysed on the fifth day in DM (DM5).

-Primary human myoblasts and treatment

Primary human myoblasts were extracted from fresh muscle biopsies of adult healthy subjects undergoing orthopedic surgery intervention. According to Italian law, institutional review board approval was not required for the use of human tissue removed during surgical procedures. Patients gave approval for intraoperative muscle biopsy and data publication. Biopsies were mechanically and enzymatically dissociated, using solutions of collagenase/dispase and collagenase type II (Sigma-Aldrich). Cells were grown on collagen-I coated plates and maintained in Growth Medium (GM), containing RPMI medium supplemented with 20% FBS (Sigma Aldrich) and 0.5 ng/ml HGF (Gibco). Human muscle cells were treated with 50 μg/ml of EVs isolated from serum of healthy subjects and patients with alcohol-related cirrhosis at the time of their shift into differentiation medium (DM0) and after 3 days (DM3) of differentiation. Finally, they were analysed on the fifth day in DM (DM5).

-Extracellular vesicles staining and EVs uptake

To assess EV uptake and the transfer of EV cargo in C2C12 cell cultures, extracellular vesicles isolated from murine serum or liver samples were co-stained with a PKH26 Red Fluorescent Cell Linker Kit (Sigma-Aldrich) and with SYTO® RNA select green fluorescent cell stain (ThermoFisher). EV staining was performed following the manufacturer's instructions. Afterwards, Exosome Spin Columns (MW 3000) (Life Technologies) were used to eliminate the dyes not bound to EVs, according to the manufacturer's protocol. Thus, C2C12 cells were exposed for 3 hours to $PKH26^{+}/Syto^{+}$- labelled EVs isolated from serum or liver of CD- and EtO-mice. To assess the effects of more prolonged exposure, EVs were stained with only the PKH26 dye and C2C12 cells were exposed to them for 24 hours.

Human primary muscle cells similarly were treated using this condition; therefore, they were exposed to $PKH26^{+}$- labelled EVs from cirrhotic patients and healthy subjects for 24 hours.

In all experiments, after EV exposure, cells were washed and fixed with 4% paraformaldehyde (PFA) and subsequently were visualized using an inverted microscope (Axioskop 2 plus; Carl Zeiss MicroImaging, Inc.) or a fluorescence laser scanning confocal microscope Leica Laser Scanning TCS SP2.

-Nanoparticle Tracking Analysis (NTA) of EVs

Serum-derived EVs, purified from human and murine samples, were resuspended to a final volume of 1 ml in PBS solution (dilution 1:500) and analysed by NanoSight NS300 instrument (Malvern Panalytical), using the NanoSight NTA software and following the manufacturer's instructions. NTA provided information about amount of particles/ml and EV size, for each analysed sample**.**

-miRNAs cell transfection

C2C12 cells (obtained from ATCC) were cultured to 70-80% confluence in 24 well plates and transfected using Lipofectamine RNAiMAX in Opti-MEM Medium (ThermoFisher) with 50 nM of Mimic miR-122 (5' – UGGAGUGUGACAAUGGUGUUUG - 3'), Mimic miR-155 (5' – UUAAUGCUAAUUGUGAUAGGGGU – 3'), Mimic miR-122+155 and miRNA Mimic Negative control. After 24h, culture medium was replaced with DM. Mimic miRNAs were made based on sequences provided by ThermoFisher (assay ID MC11012 and MC13058) and supported by miRbase database. After 72h, cells were collected for analyses.

-Protein extraction and Western Blot

Protein extraction was performed by homogenizing cells, EVs and tissue samples in Ripa lysis buffer, containing protease and phosphatase inhibitors (Sigma-Aldrich) [8]. Protein extracts were quantified using the Bradford Protein Assay (Bio-Rad, Inc), and equal amounts of protein from each lysate were separated on an SDS polyacrylamide gel (4-15% Criterion™ TGX Stain-Free™ Precast gel, Bio-Rad). Proteins were transferred into a nitrocellulose membrane (Trans-Blot Turbo transfer pack, Bio-rad) using the Trans-Blot Turbo™. Transfer System. Filters were then blocked with 5% BSA in TBS-1% Tween for 1h at RT, followed by an incubation with a primary antibody in blocking solution overnight at 4°C. Membranes were incubated with the following antibodies: p-mTOR (Ser2448), mTOR, p-AKT (Ser473), AKT, p-GSK-3β (Ser9); GSK3, ATG5, LC3B (Cell Signaling). EVs isolated from serum and liver tissue of mice were analyzed using following antibodies: CD9 (System Biosciences); CD81(Novusbio) and ASGR1 (LifeSpan BioSciences), while human EVs were analyzed using CD63 (System Biosciences) and Alix (Santa Cruz Biotechnology, Inc). After washing in TBS-1% Tween, the membrane was incubated with a specific peroxidase-conjugated secondary antibody and then visualized by the enhanced chemiluminescence system (ChemiDoc Imaging System, Bio-Rad). The acquired signal was evaluated by scanning densitometry using a bio-image analysis system (Image Lab™ Software). The results were expressed as ratio of relative intensities of p-mTOR/mTOR, p-AKT/AKT, p-GSK3/GSK3 and LC3B-II/I, while ATG5 protein levels were measured normalizing Western blot band intensity to stain-free total lane protein. The expression levels of all analyzed proteins were calculated with respect to CD-group and reported as mean fold change values. Stain free or Ponceau were used as a loading control

-Histological analysis

Hematoxylin and Eosin staining

Muscle and liver tissues from CD- and EtOH- mice groups were embedded in tissue freezing medium and snap-frozen in nitrogen-cooled isopentane. For histological and morphometric analysis, frozen cross-sections (7 µm) from the middle region of muscle and liver were stained with hematoxylin and eosin [8]. Images were taken using AxioVision 3.1 software (Carl Zeiss). Cross-sectional area (CSA) of muscle fibers was analyzed with ImageJ software.

Oil red O staining

Oil red staining was performed according to a standard procedure. Oil Red stock solution was prepared by dissolving 175 mg of Oil Red O (Sigma Aldrich) in 50 ml of isopropanol (Sigma Aldrich). The solution was filtered and diluted to obtain working solution. Liver cryosections were fixed for 1h with 10% formalin solution (Sigma Aldrich) at 4°C. After rinsing with water, sections were incubated for 10 minutes with 60% isopropanol, then for 15 minutes with ORO working solution. Finally, slides were mounted with 10% glycerol.

-Immunofluorescence analysis on liver tissue and muscle cells

Frozen sections (7-10 µm) of liver tissue from CD- and EtOH- mice were fixed with 4% PFA, washed in BSA blocking buffer (1% filtered BSA and 0.2% Triton x-100 in PBS), incubated in 10% goat serum for 1 hour and then incubated overnight at 4°C with F4/80 antibody (Cell Signaling). Afterwards, samples were washed in PBS with 0.2% TritonX-100 and incubated with a secondary antibody (Life Technologies) for 1h at RT.

Immunofluorescence analysis on muscle cell culture was performed using the same protocol [8]. Thus, C2C12 cells or murine and human primary muscle cells were fixed with 4% PFA and incubated overnight at 4°C with a primary antibody

against MyHC (MF20 Hybridoma Bank). Finally, tissue sections and cells nuclei were stained with Hoechst (Sigma Aldrich), mounted in standard mounting media and imaged by fluorescence microscopy (Axioskop 2 plus; Carl Zeiss) or a fluorescence laser scanning confocal microscope Leica Laser Scanning TCS SP2.

For analysis of muscle cell culture, fusion index was determined as a percentage of the number of nuclei in differentiated myotubes over the total number of nuclei (5-9 microscopic fields of three independent cultures, magnification 10x). All morphometrical analysis were performed by ImageJ software.

Supplementary Figures

Figure S1

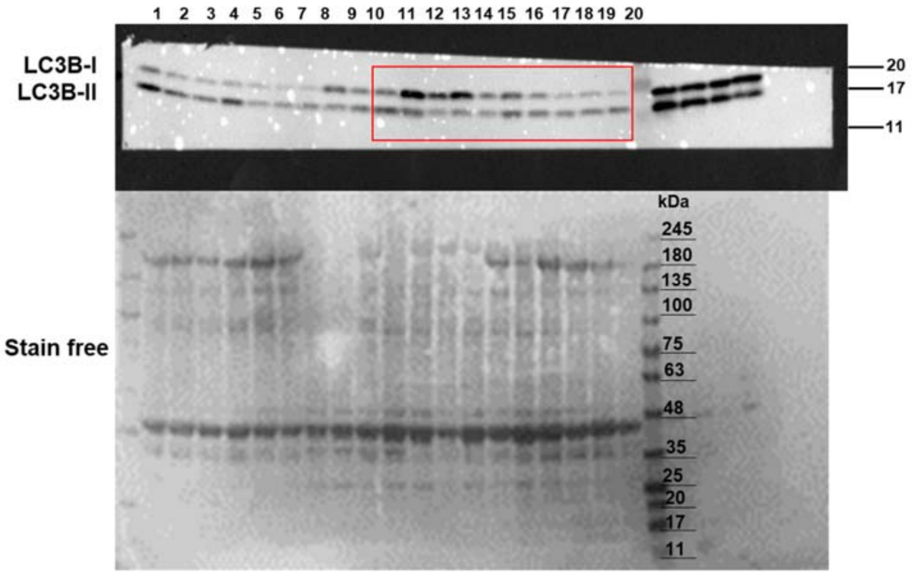

Figure S1. Full-length Western blot images for Figure 2F. Full-length western blot images for the detection of LC3B-I and LC3B-II proteins. From lane 10 to 14: protein extracts of gastrocnemius of CD-mice. From lane 15 to 19: protein extracts of EtOH-mice. Lane 20: Prestained marker (Enzo’s Prestained Protein Ladder).

Figure S2

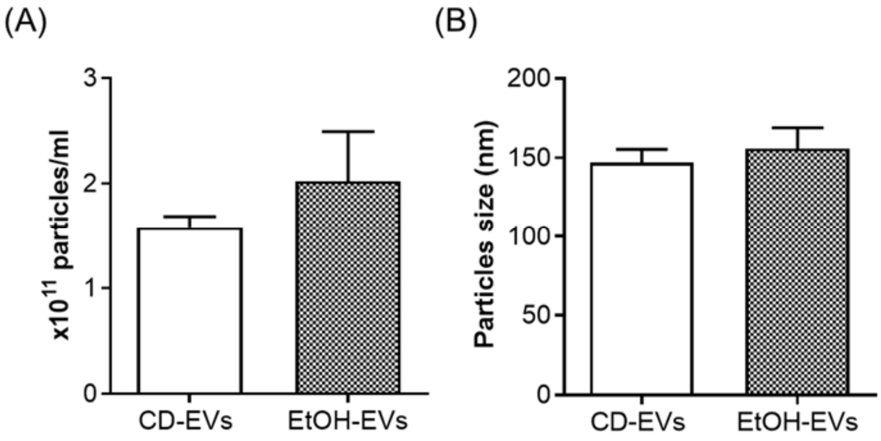

Figure S2. Characterization of isolated extracellular vesicles from serum of CD and EtOH-mice by Nanoparticle tracking analysis. Plots showing (A) number of EVs (particles per ml of serum) and (B) EV mean particle size (nm) (n=4). All data are expressed as mean ± SEM.

Figure S3

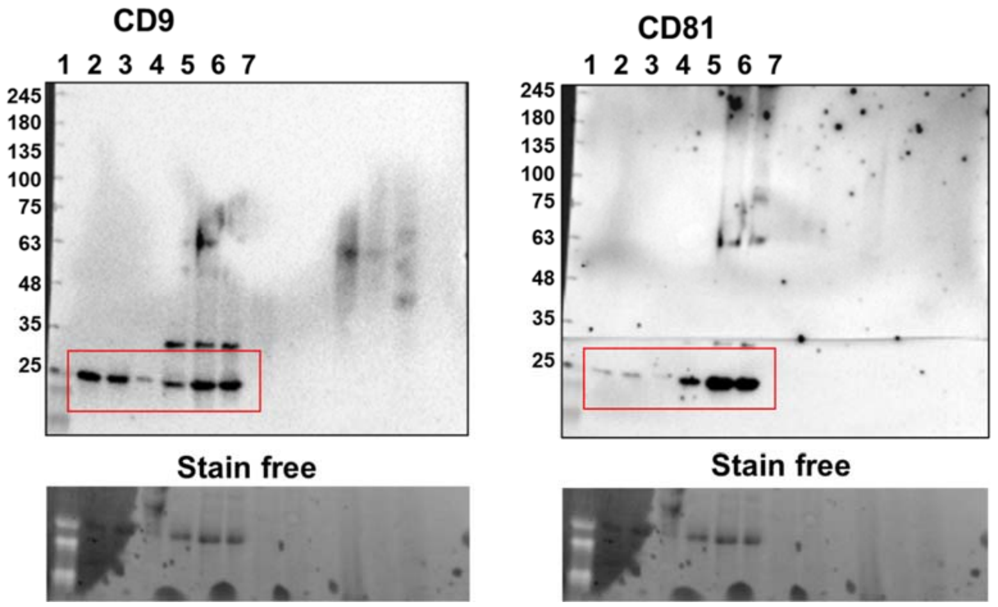

Figure S3. Full-length Western blot images for Figure 3B. Full-length western blot images for the detection of CD9 and CD81 proteins. Lane 1: Prestained marker (Enzo's Prestained Protein Ladder). From lane 2 to 4: serum EVs isolated from CD-mice. From lane 5 to 7: serum EVs isolated from EtOH-mice.

Figure S4

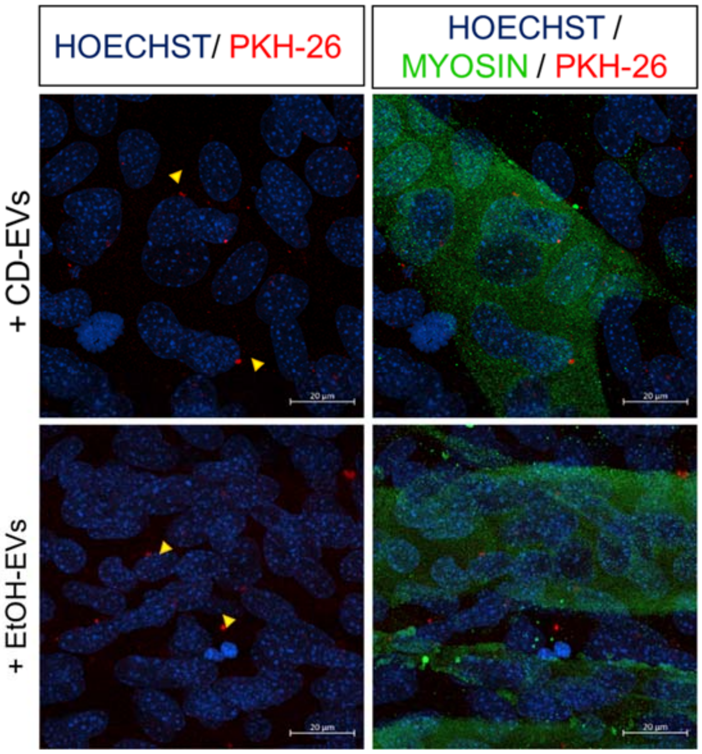

Figure S4. Representative confocal microscopy images of C2C12 cell culture exposed for 24h with PKH-26 labeled serum EVs; arrowheads indicate red stained EVs; nuclei are stained in blue and myosin positive myotubes are stained in green (scale bar: 20μm).

Figure S5

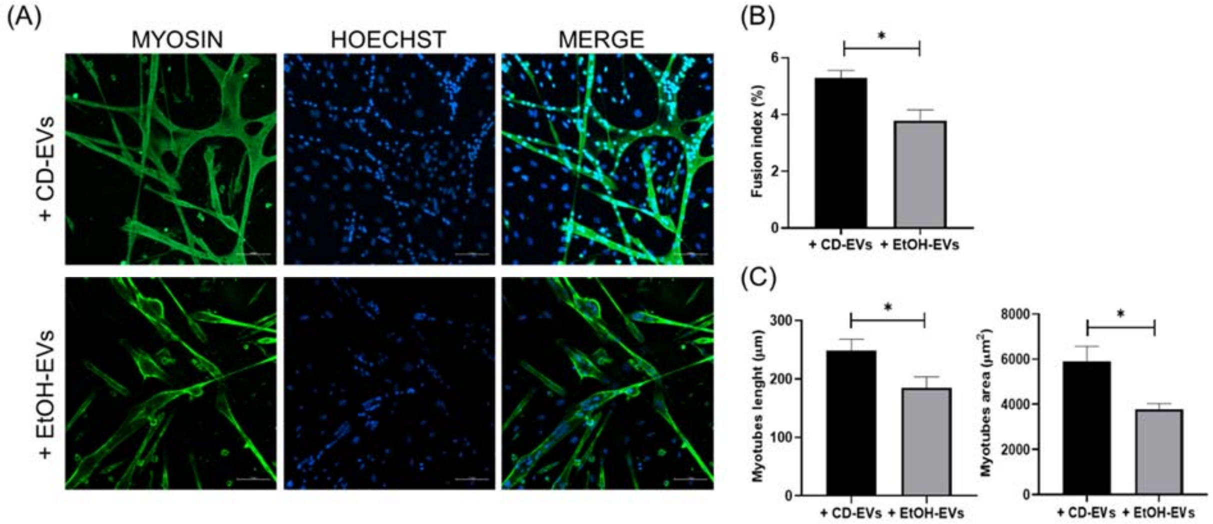

Figure S5. Analysis of primary culture of mouse skeletal muscle cells (MuSCs) exposed to EVs isolated from serum of CD- and EtOH-mice. (A) Representative images of immunofluorescence analysis for Myosin on muscle cell culture at 5 days of differentiation (DM5), after exposure to serum EVs derived from CD- and EtOH-mice. EVs exposure was performed at time of the shift in differentiation medium (DM0) and after 3 days (DM3) (scale bar: 100 µm). (B) Measurement of Fusion index and (C) morphometric analysis of myotubes in EVs-treated muscle cell culture at DM5. Nuclei and myotubes were examined in 4-9 microscopic fields (scale bar: 100µm) for each group in three independent cultures. All data are expressed as mean ± SEM. Data were analyzed by Mann–Whitney U-test. Muscle cell culture treated with EtOH-EVs versus that treated with CD-EVs, *$p<0.05$.

Figure S6

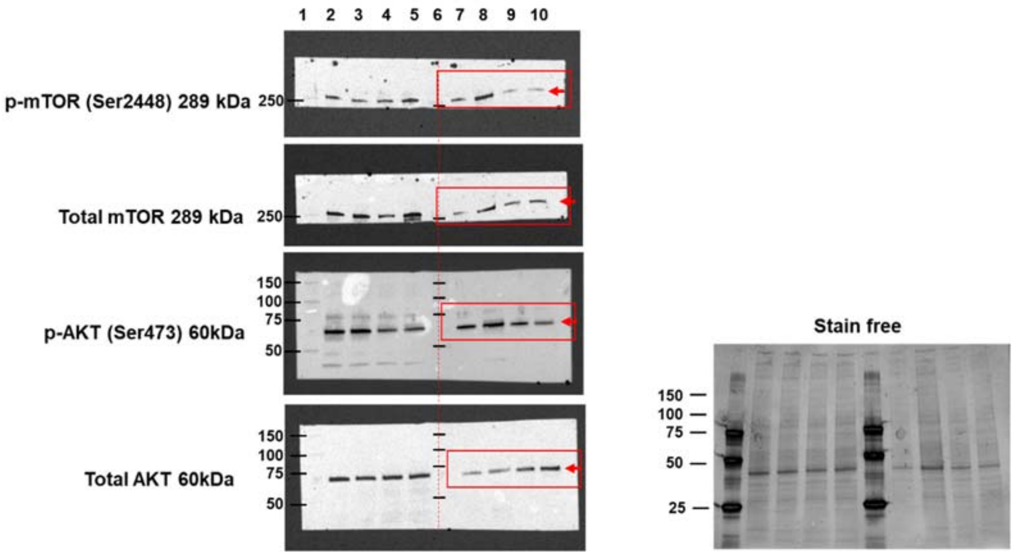

Figure S6. Full-length Western blot images for Figure 4D. Full-length western blot images for the detection of p-mTOR (Ser2448), Total m-TOR, p-AKT (Ser473) and Total AKT proteins. Lane 1 and 6: Prestained marker (Precision Plus Protein^{TM} Western C^{TM} Blotting standards, Biorad). From lane 7 to 8: C2C12 cells + CD-EVs. From lane 9 to 10: C2C12 cells + EtOH-EVs.

Figure S7

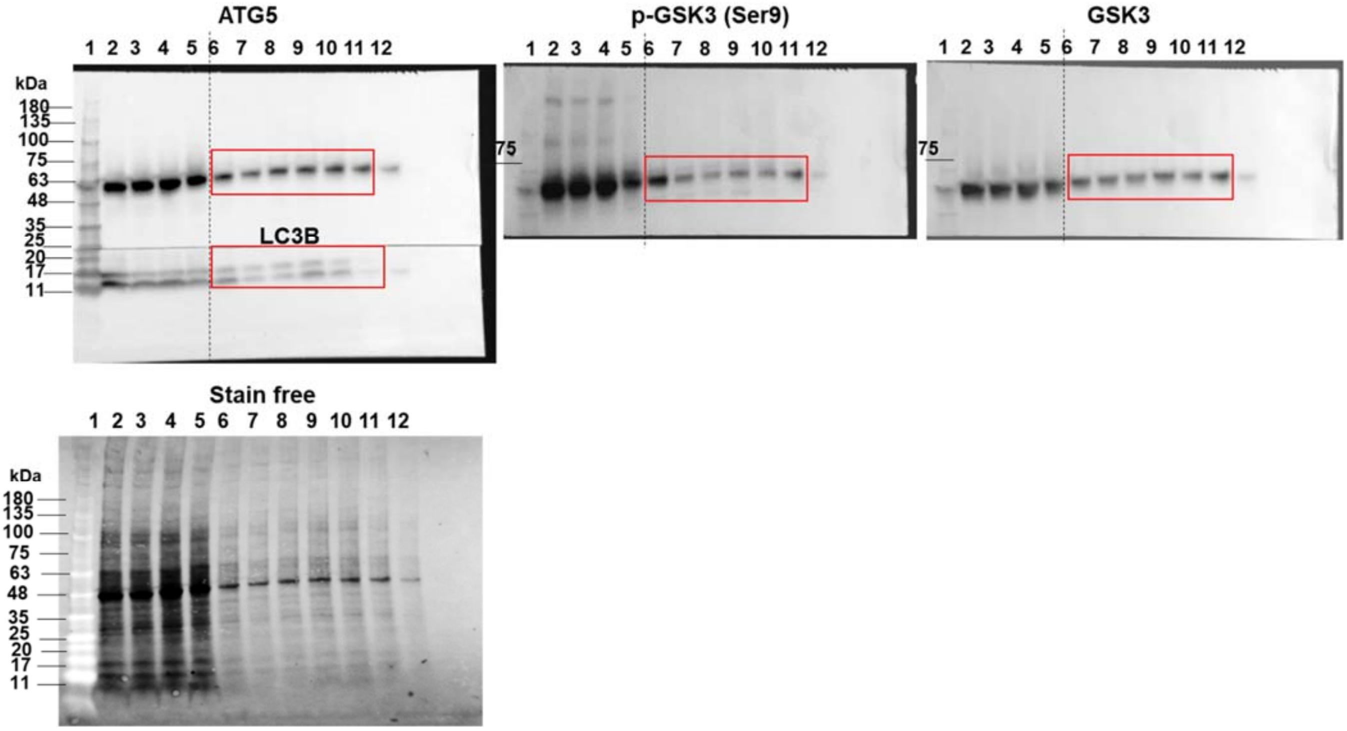

Figure S7. Full-length Western blot images for Figure 4E. Full-length western blot images for the detection of ATG5, p-GSK3(Ser9) and GSK3 proteins. Lane 1: Prestained marker (Enzo's Prestained Protein Ladder). From lane 6 to 8: C2C12 cells + CD-Evs. From lane 9 to 10: C2C12 cells + EtOH-Evs.

Figure S8

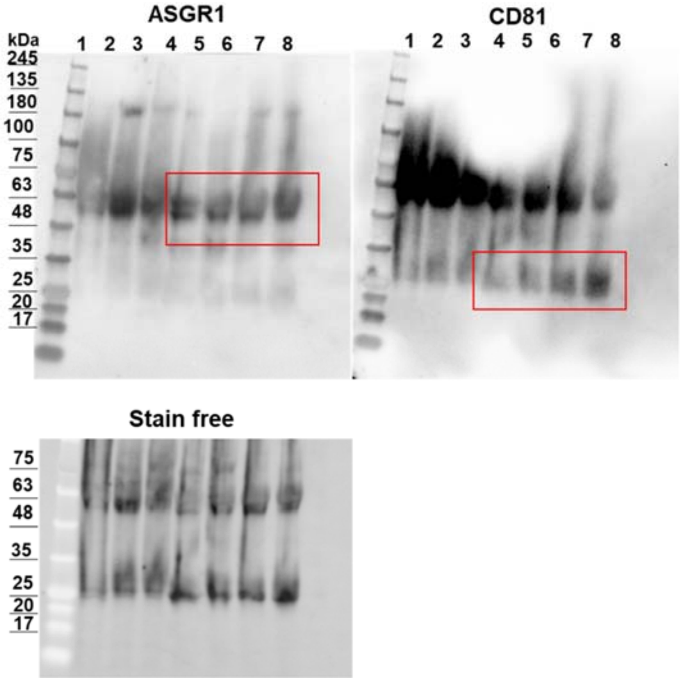

Figure S8. Full-length Western blot images for Figure 5A. Full-length western blot images for the detection of ASGR1 and Cd81 proteins**.** Lane 1: Prestained marker (Enzo's Prestained Protein Ladder). From lane 5 to 6: Hepatic EVs of CD-mice. From lane 7 to 8: Hepatic EVs of EtOH-mice.

Figure S9

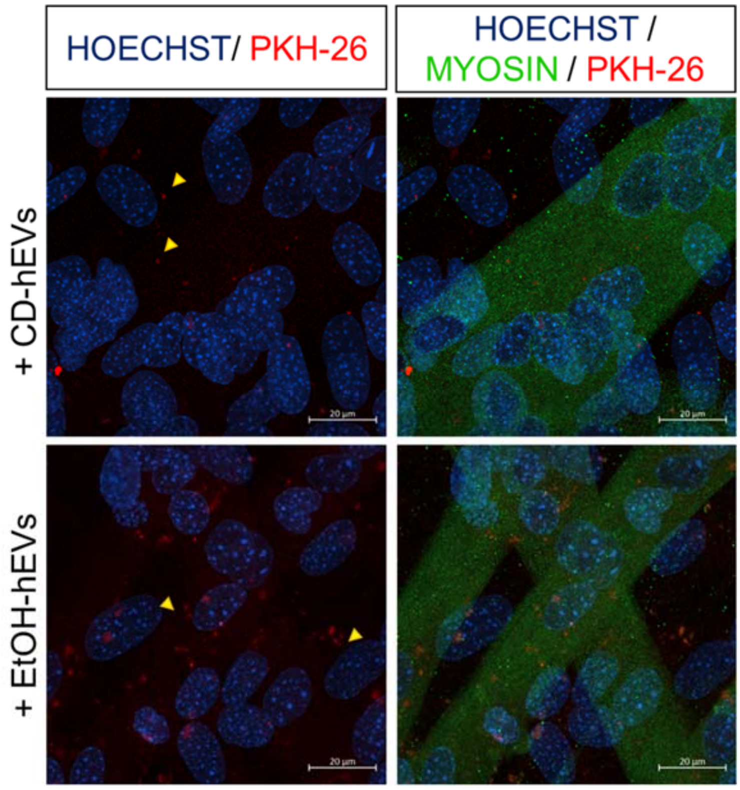

Figure S9. Representative confocal microscopy images of C2C12 cell culture exposed for 24h to PKH-26 labeled hepatic EVs; arrowheads indicate red stained hepatic EVs; nuclei are stained in blue and myosin positive myotubes are stained in green (scale bar: 20µm).

Figure S10

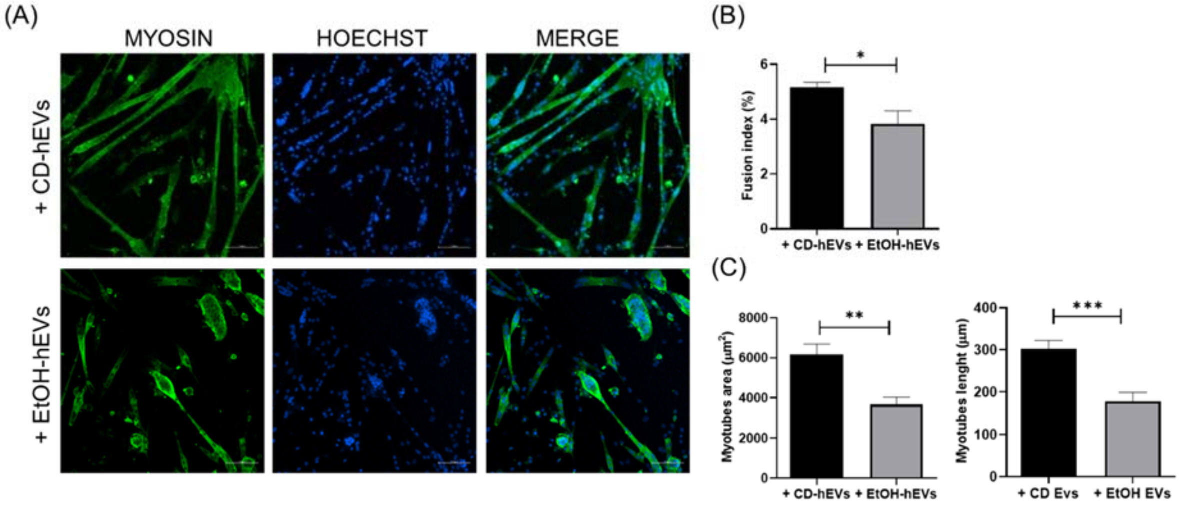

Figure S10. Analysis of primary culture of mouse skeletal muscle cells (MuSCs) exposed to hepatic Evs (hEVs) isolated from liver of CD- and Et-OH mice. (A) Representative images of immunofluorescence analysis for Myosin on mouse skeletal muscle cells, at 5 days of differentiation (DM5), exposed to hepatic EVs (hEVs) derived from CD and EtOH-mice; hEVs exposure was performed at at time of the shift in differentiation medium (DM0) and after 3 days (DM3) (scale bar: 100 µm). (B) Measurement of fusion index and (C) morphometric analysis of myotubes in hEVs-treated MuSCs cell culture at DM5. Nuclei and myotubes were examined in 4-9 microscopic fields (scale bar: 100µm) for each group in three independent cultures. All data are expressed as mean ± SEM. Data were analyzed by Mann–Whitney U-test. Muscle cell culture treated with EtOH-hEVs versus that treated with CD-hEVs, *p<0.05, **p<0.01 and ***p<0.001.

Figure S11

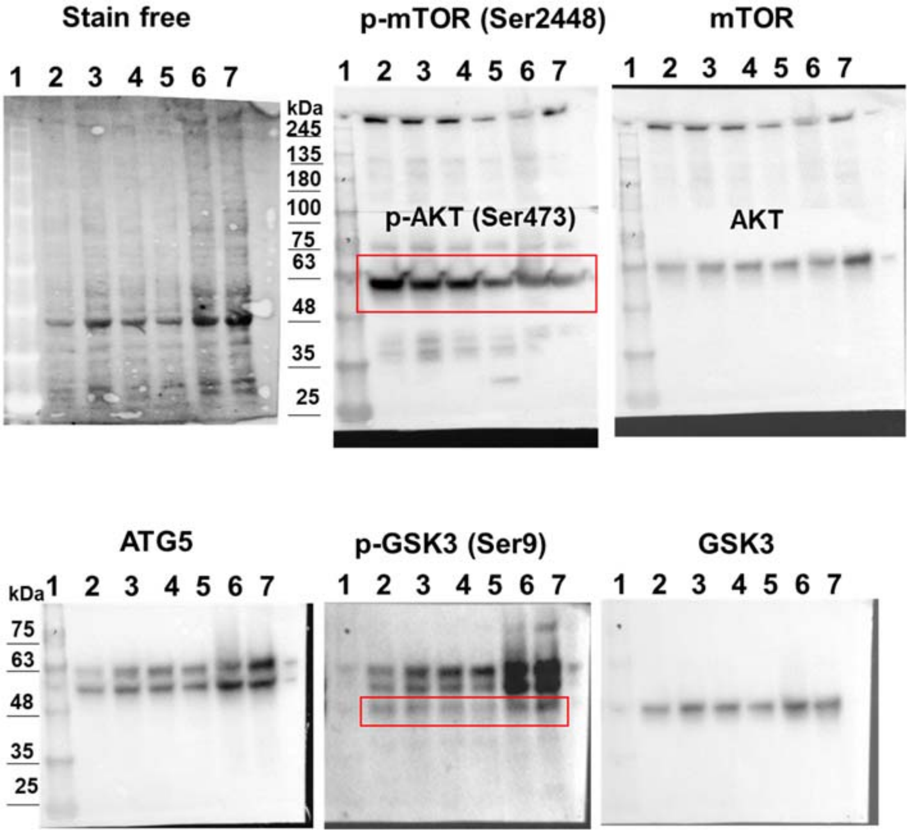

Figure S11. Full-length Western blot images for Figure 6E. Full-length western blot images for the detection of p-mTOR(Ser2448), mTOR, ATG5, p-GSK3(Ser9) and GSK3 proteins. Full-length Western blot images. Full-length blot image for Figure 6E. Lane 1: Prestained marker (Enzo's Prestained Protein Ladder). From Lane 2 to 4: C2C12 cells + CD-hEVs. From Lane 5 to 7: C2C12 cells + EtOH-hEVs.

Figure S12

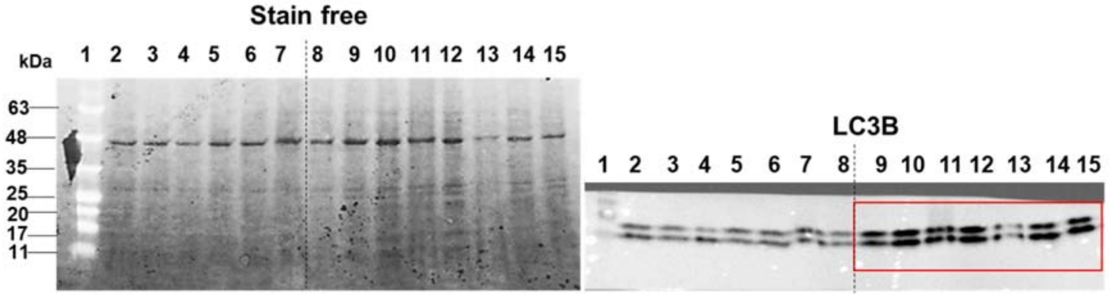

Figure S12. Full-length Western blot images for Figure 6F. Full-length western blot images for the detection ofLC3B. Lane 1: Prestained marker (Enzo's Prestained Protein Ladder). From Lane 9 to 11: C2C12 cells + CD-hEVs. From Lane 12 to 15: C2C12 cells + EtOH-hEVs.

Figure S13

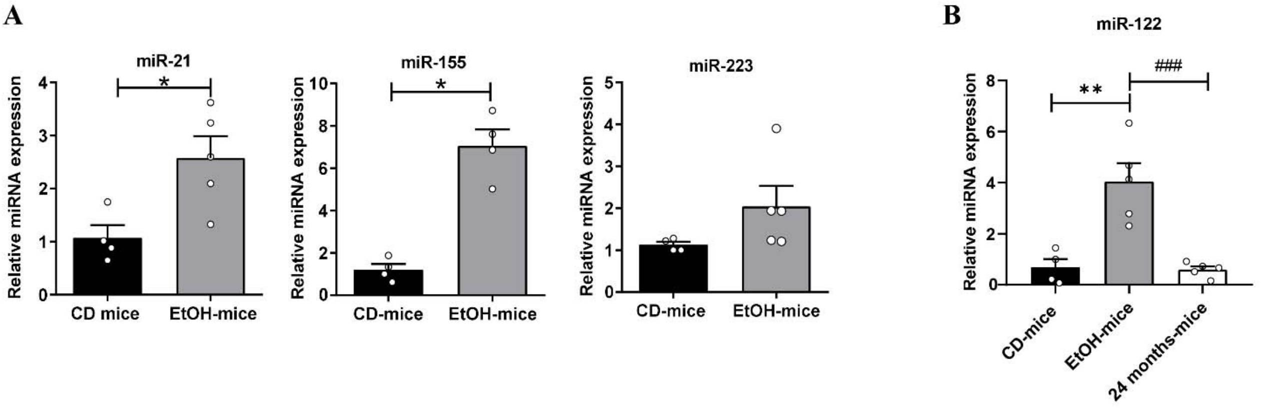

Figure S13. miRNAs expression in skeletal muscle. (A) Real-time PCR analysis to evaluate miR-21, miR-155 and miR-223 expression levels in gastrocnemius muscle of CD- and EtOH-mice (n≥4). All data are expressed as the mean ± SEM. Data were analyzed by Mann–Whitney U-test. EtOH mice versus CD mice, *p<0.05. (B) Real-time PCR analysis to evaluate miR-122 expression levels in gastrocnemius muscle of CD-mice, EtOH-mice and 24-month-old mice (n≥4). All data are expressed as the mean ± SEM. Statistical analysis was performed with One-way ANOVA and Turkey's multiple comparison test. EtOH mice versus CD mice, **p<0.01; 24 months-mice vs EtOH-mice ###p<0.001.

Figure S14

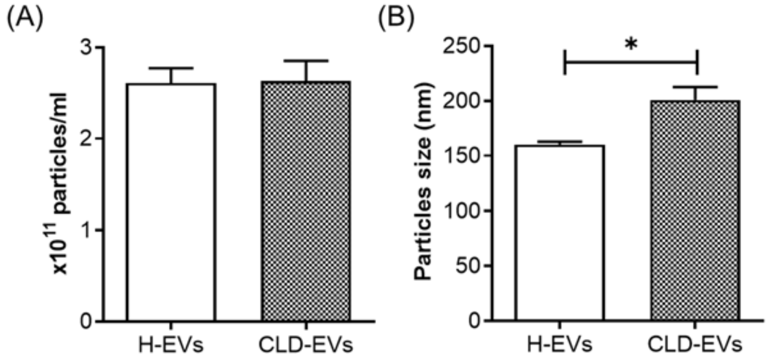

Figure S14. Characterization of isolated extracellular vesicles from serum of healthy subjects and cirrhotic patients (CLD) by Nanoparticle tracking analysis. Plots showing (A) concentration of EVs (number of particles per ml of serum) and (B) EV mean particle size (nm) (n=4). All data are expressed as mean ± SEM.

Figure S15

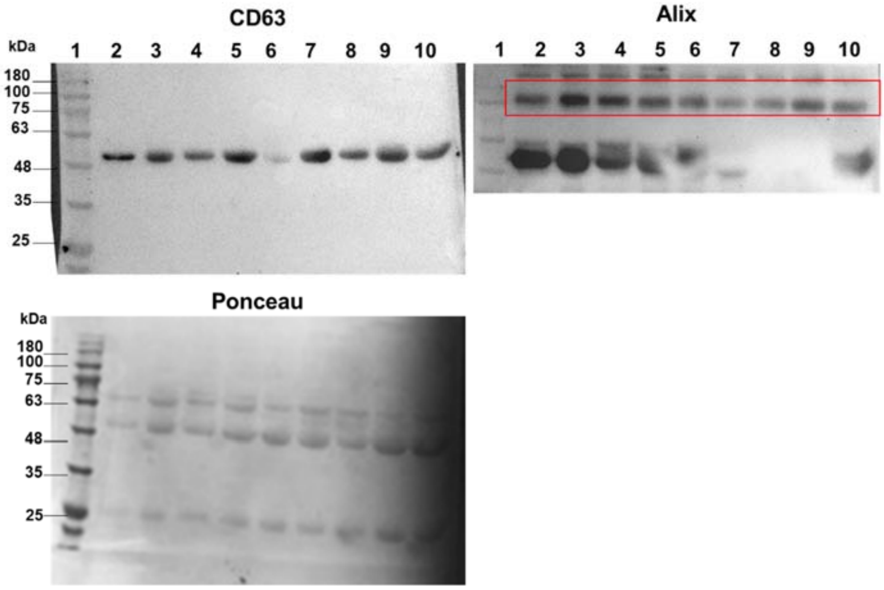

Figure S15. Full-length Western blot images for Figure 8A (right panel). Full-length western blot images for the detection of CD63 and Alix proteins. Lane 1: Prestained marker (Enzo's Prestained Protein Ladder). From lane 2 to 5: Serum EVs of healthy subjects. Lane 6 to 10: Serum EVs of CLD patients.

Figure S16

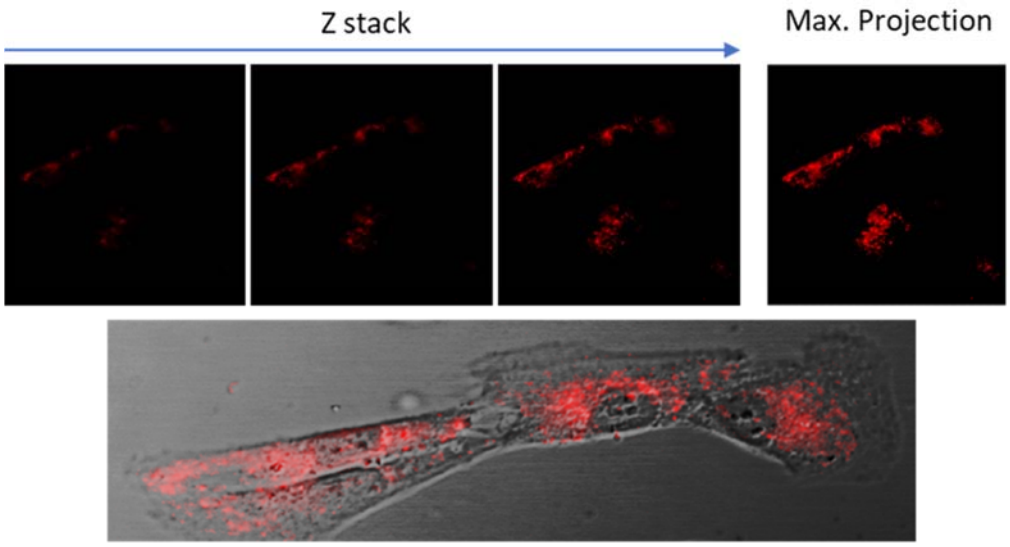

Figure S16. Representative confocal microscopy images of human muscle cells exposed 24h with PKH26- labeled EVs. Z-stack images at sequential focal planes 0.5 µm apart (20x objective, with a 4x digital zoom).

Figure S17

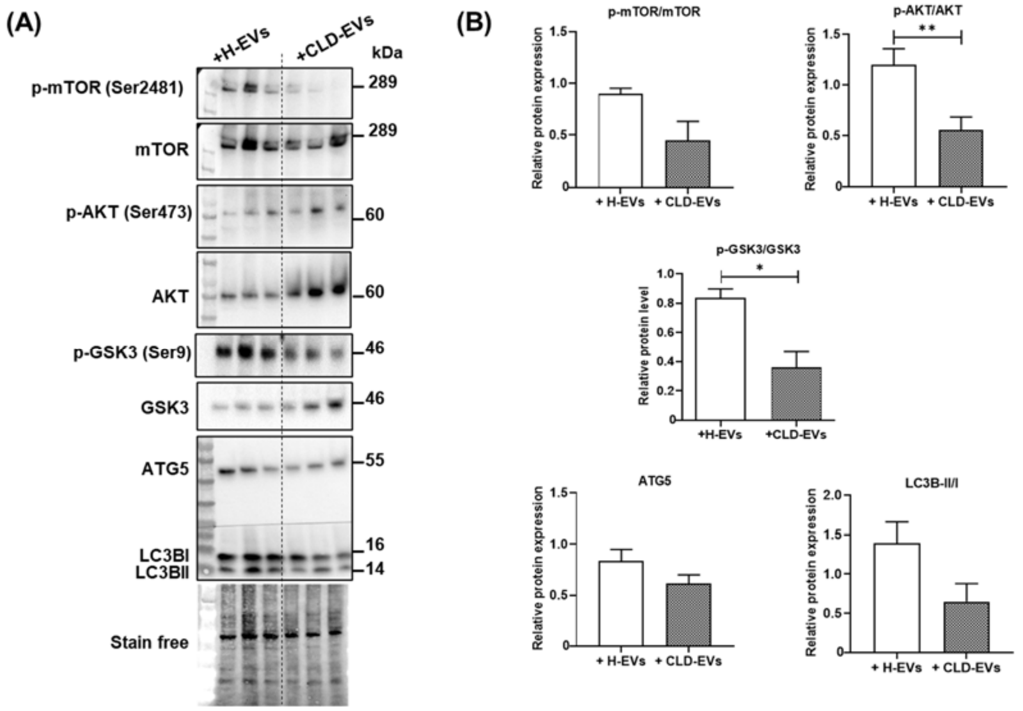

Figure S17. *In vitro* treatment of human muscle cell culture with circulating EVs isolated from serum samples of healthy individuals (H-EVs) and cirrhotic patients (CLD-EVs) induced muscle atrophy. Human primary skeletal muscle cells were exposed to circulating EVs at the start of differentiation (DM0) and after 3 days (DM3). Representative images (left panel) and densitometric analysis (right panels) of Western blot for p-mTOR (Ser2481), total mTOR, p-AKT (Ser473), total AKT, p-GSK3 (Ser9), total GSK3, ATG5 and LC3B-I and LC3B-II proteins in HD- and CLD-treated human muscle culture at DM5. Protein expression levels were expressed as ratio of relative intensities of p-mTOR/mTOR, p-AKT/AKT, p-GSK3/GSK3 and LC3B-II/I; (n≥5 per group), while ATG5 protein levels were measured normalizing Western blot band intensity to stain-free total lane protein (n≥5 for each group in three independent cultures). Stain free was used as a loading control. All data are expressed as mean ± SEM. Data are analyzed by Mann–Whitney U-test. Human muscle culture treated with CLD-EVs versus culture treated with H-EVs, *$p<0.05$.

Figure S18

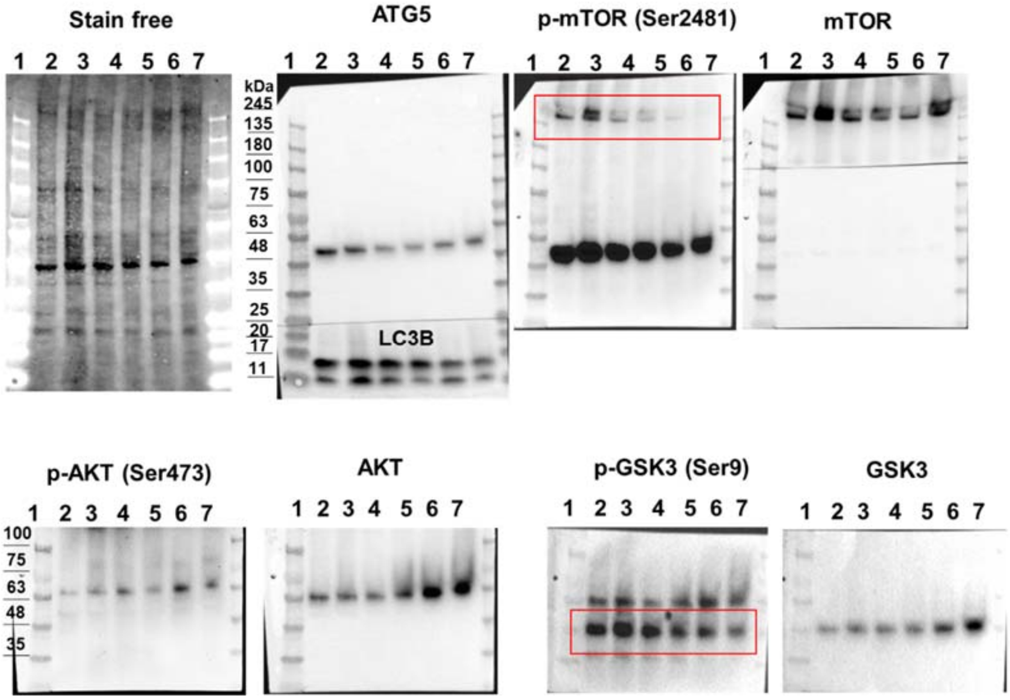

Figure S18. Full-length Western blot images for Figure S17 (left panel). Full-length western blot images for the detection of ATG5, p-mTOR(Ser2481), mTOR, p-AKT(Ser473), AKT, p-GSK3(Ser9) and GSK3 proteins. Full-length Western blot images. Full-length blot image for Figure 8A (right panel). Lane 1: Prestained marker (Enzo's Prestained Protein Ladder). From lane 2 to 4: Human primary skeletal muscle cells + serum H-EVs Lane 7 to 8: Human primary skeletal muscle cells + serum CLD-Evs.

Additional references

Ref S1. Nath B, Levin I, Csak T, Petrasek J, Mueller C, Kodys K, Catalano D, Mandrekar P, Szabo G. Hepatocyte-specific hypoxia-inducible factor-1α is a determinant of lipid accumulation and liver injury in alcohol-induced steatosis in mice. Hepatology. 2011 May;53(5):1526-37.

Ref S2. Mandrekar P, Ambade A, Lim A, Szabo G, Catalano D. An essential role for monocyte chemoattractant protein-1 in alcoholic liver injury: regulation of proinflammatory cytokines and hepatic steatosis in mice. Hepatology. 2011; 54:2185–2197.

Ref S3. Kusumanchi P, Liang T, Zhang T, Ross RA, Han S, Chandler K, Oshodi A, Jiang Y, Dent AL, Skill NJ, Huda N, Ma J, Yang Z, Liangpunsakul S. Stress-Responsive Gene FK506-Binding Protein 51 Mediates Alcohol-Induced Liver Injury Through the Hippo Pathway and Chemokine (C-X-C Motif) Ligand 1 Signaling. Hepatology. 2021;74(3):1234-1250.

Ref S4. Mirzoev TM, Sharlo KA, Shenkman BS. The Role of GSK-3β in the Regulation of Protein Turnover, Myosin Phenotype, and Oxidative Capacity in Skeletal Muscle under Disuse Conditions. Int J Mol Sci. 2021 May 11;22(10):5081.

Ref S5. Li G, Luo W, Abdalla BA, Ouyang H, Yu J, Hu F, Nie Q, Zhang X. miRNA-223 upregulated by MYOD inhibits myoblast proliferation by repressing IGF2 and facilitates myoblast differentiation by inhibiting ZEB1. Cell Death Dis. 2017 Oct 5;8(10):e3094.
